# Altered DNA methylation associated with an abnormal liver phenotype in a cattle model with a high incidence of perinatal pathologies

**DOI:** 10.1038/srep38869

**Published:** 2016-12-13

**Authors:** Hélène Kiefer, Luc Jouneau, Évelyne Campion, Delphine Rousseau-Ralliard, Thibaut Larcher, Marie-Laure Martin-Magniette, Sandrine Balzergue, Mireille Ledevin, Audrey Prézelin, Pascale Chavatte-Palmer, Yvan Heyman, Christophe Richard, Daniel Le Bourhis, Jean-Paul Renard, Hélène Jammes

**Affiliations:** 1 UMR BDR, INRA, ENVA, Université Paris Saclay, 78350, Jouy en Josas, France; 2 INRA, UMR0703 APEX, Oniris, Nantes, France; 3 UMR MIA-Paris, AgroParisTech, INRA, Université Paris-Saclay, 75005, Paris, France; 4 Institute of Plant Sciences Paris Saclay IPS2, CNRS, INRA, Université Paris-Sud, Université Evry, Université Paris-Saclay, 91405 Orsay, France; 5 Institute of Plant Sciences Paris-Saclay IPS2, Paris Diderot, Sorbonne Paris-Cité, Bâtiment 630, 91405, Orsay, France; 6 INRA, UE1298, Unité Commune d’Expérimentation Animale, Leudeville, France

## Abstract

Cloning enables the generation of both clinically normal and pathological individuals from the same donor cells, and may therefore be a DNA sequence-independent driver of phenotypic variability. We took advantage of cattle clones with identical genotypes but different developmental abilities to investigate the role of epigenetic factors in perinatal mortality, a complex trait with increasing prevalence in dairy cattle. We studied livers from pathological clones dying during the perinatal period, clinically normal adult clones with the same genotypes as perinatal clones and conventional age-matched controls. The livers from deceased perinatal clones displayed histological lesions, modifications to quantitative histomorphometric and metabolic parameters such as glycogen storage and fatty acid composition, and an absence of birth-induced maturation. In a genome-wide epigenetic analysis, we identified DNA methylation patterns underlying these phenotypic alterations and targeting genes relevant to liver metabolism, including the type 2 diabetes gene *TCF7L2*. The adult clones were devoid of major phenotypic and epigenetic abnormalities in the liver, ruling out the effects of genotype on the phenotype observed. These results thus provide the first demonstration of a genome-wide association between DNA methylation and perinatal mortality in cattle, and highlight epigenetics as a driving force for phenotypic variability in farmed animals.

Although there is increasing evidence that epigenetics participates in phenotypic variability in many species, epigenetic data underlying complex traits are scarce in livestock^1^, in part because epigenetic factors are confounded with the effects of DNA sequence polymorphism^2^. One example of complex traits with increasing prevalence in dairy cattle is perinatal mortality^3^, which is an important issue for animal welfare, and is also detrimental to both milk production and maternal health and fertility. Since perinatal mortality involves both genetic (breed of the dam, genetic merit of the sire) and non-genetic (herd management, breeding method) factors, cloned animals with identical genotypes but different developmental abilities can be used to demonstrate the association between epigenetic factors and this trait. The production of clones from an adult somatic cell is enabled by the capacity of the oocyte to reprogramme the epigenome of a differentiated nucleus to totipotency, and aberrant epigenetic reprogramming is recognized as one of the leading causes of the developmental abnormalities encountered in clones^4^. Accordingly, DNA methylation defects have been reported in bovine clones, at both the global level^5^,^6^ and at candidate loci^7^,^8^,^9^, but genome-wide views of the affected regions are still scarce^10^ and associations with phenotypic anomalies have not been clearly demonstrated.

Despite important advances in the procedures^11^,^12^,^13^,^14^, only 11.5% of cloned embryos transferred to recipient cows develop to term^15^. As well as the constant developmental waste observed after implantation in cloned pregnancies^16^, the perinatal period is marked by important foetal and neonatal losses^17^,^18^,^19^. Foetal growth peaks towards the end of pregnancy and makes considerable demands on the maternal nutrient supply. The impaired placental function observed in cloned pregnancies may hamper adequate exchanges between the dam and foetus, resulting in these important perinatal losses and in metabolic disorders at the start of postnatal life^20^. Although the precise cause of death is unclear in most cases, large offspring syndrome and large placenta, which are often associated with *hydrops fetalis* and maternal hydrallantois, have frequently been observed in a context of clone stillbirth^21^. Episodic losses are still reported up to six months of age, after which the health of clones is compatible with reproduction, lactation and meat production in most animals^22^,^23^,^24^,^25^. To date, adult clones have been considered as being free of major abnomalities^26^, suggesting a selection of normal individuals through the gradual loss of pathological clones during development^27^. The large number of abnormalities observed in perinatal clones, and the clinically normal phenotype of adult clones arising from the same donor cells, thus provide an unique opportunity to explore the DNA sequence-independent mechanisms underlying perinatal mortality in cattle.

Here we examined whether the phenotypic defects linked to perinatal mortality in clones could be associated with altered DNA methylation profiles. The liver was selected for this study because it displays a grossly abnormal appearance (excessive size, enlarged lymphatic vessels, steatosis) at the necropsy of deceased clones^28^,^29^, and is affected by deviations in the transcriptome^30^ and by DNA methylation defects at various developmental stages^5^,^7^. Furthermore, the liver plays a fundamental role in the metabolic transition to postnatal life, through its ability to synthesize, store and export nutrients such as glucose and lipids^31^. This ability enables the adaptation from a continuous supply of nutrients *in utero* to enteral feeding after birth, which is precisely the stage through which many clones are unable to pass. We performed a qualitative and quantitative analysis of the liver structure and composition in pathological perinatal clones (both foetuses and neonates), clinically normal adult clones with the same genotypes as the perinatal deceased clones, and age-matched controls obtained by artificial insemination (AI). We report here on histomorphometric and metabolic parameters that were specifically altered in the livers of pathological perinatal clones, and which might therefore represent markers of the hepatic pathologies encountered in cloned animals. Finally, we identified DNA methylation patterns correlated to these phenotypic markers and targeting genes relevant to hepatic metabolism. Interestingly, these epigenetic patterns were similar to those of adults, suggesting that an uncoupling of DNA methylation and age occurred in the pathological clones.

## Results

### The livers of perinatal clones display pathological features and altered histomorphometric parameters

**Figure 1:**
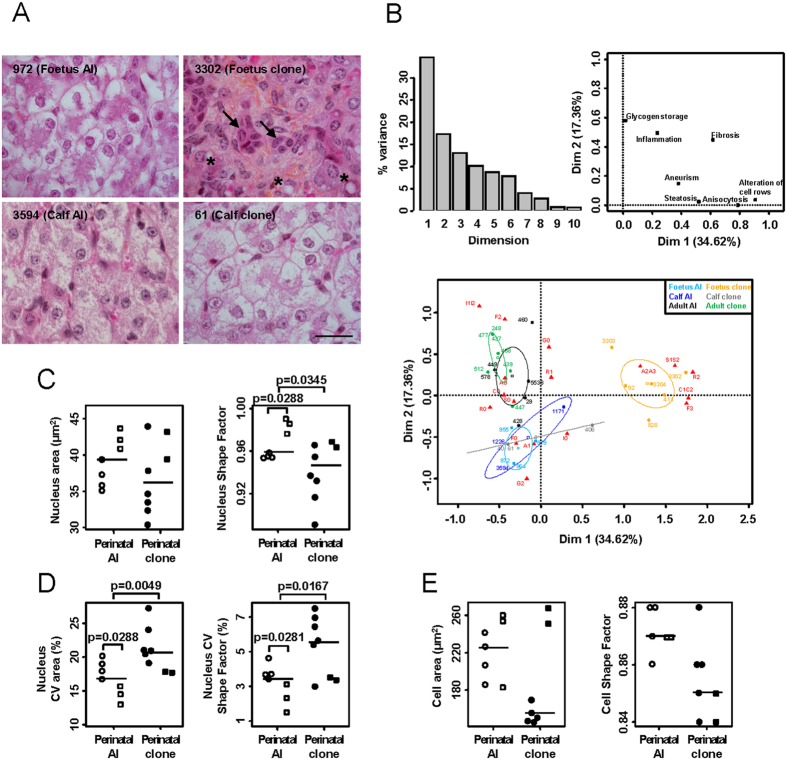
Altered histological structure of the liver in pathological perinatal clones. (**A**) Liver sections stained with haemalin-eosin-saffron are shown for one AI control foetus, one cloned foetus, one control calf and one cloned calf. Fibrotic areas are revealed by their yellow appearance following saffron staining. The arrows indicate disorganization of the rows of hepatocytes and the asterisks highlight examples of intracytoplasmic lipid vesicles corresponding to mild steatosis. Scale bar: 25 μm. (**B**) Thirty animals were graded in categories according to the severity of seven types of lesions (Tables 1 and 2), and MCA was performed. Categories containing fewer than four individuals were merged, generating G1G2, S1S2, A2A3, and I1I2. Upper left panel: barplot of the contribution of each dimension to total variance, showing a marked drop between the first and second dimensions and then smaller drops. Upper right panel: variable factor map, illustrating the contribution of each variable to dimensions 1 and 2. Lower panel: individual and category factor map, with 95% confidence ellipses shown for each group. The proximity of individuals to a category indicates that this category is more represented among these individuals than in the remaining population. (**C**,**D**) For each animal, the area and a shape factor representing circularity (1: perfect circles) were measured on at least 200 hepatocyte nuclei and the mean (**C**) and CV (**D**) were calculated. The CV is indicative of cell heterogeneity in the tissue. Each dot represents one animal. Median values are indicated by horizontal lines. Open dots: perinatal AI controls; filled dots: perinatal clones. Prenatal (circles) and postnatal (squares) animals were considered separately. Significant differences between AI and clones and between prenatal and postnatal animals are indicated (p < 0.05, permutation test). (**E**) For perinatal animals with clearly observable cell limits, the cell area and shape factor were measured on 35 hepatocytes, and the mean was calculated. The differences between AI controls and clones were not significant.

Standard histological examination of the livers of clones and AI controls revealed marked lesions in some perinatal clones, such as fibrosis, generalized anisocytosis (with some small hepatocytes and a few, large, vacuolated hepatocytes reflecting mild steatosis), and pseudo-canals of small hepatocytes. These lesions are illustrated Fig. 1A in a representative individual (upper right panel). By contrast, some other perinatal animals displayed hepatocyte trabeculae of homogeneous size, with regular sized sinusoids and marked intracytoplasmic glycogen storage appearing as white and granular matter (other panels). To identify groups of animals with similar histological features, we performed multiple correspondence analysis (MCA)^32^ on scored lesions (Tables 1 and 2). Alterations to cell row organization, anisocytosis and, to a lesser extent, fibrosis and steatosis, contributed strongly to dimension 1 which explained 35% of total variance (Fig. 1B, upper panels). Dimension 1 combined pathological features that appeared more frequently in the cloned foetuses, with the frequent occurrence of categories R2 (no visible trabeculae), C1C2 (anisocytosis with some atypical cells), F3 (marked bridging fibrosis), S1S2 (moderate to important steatosis) and A2A3 (aneurysm) in these animals (lower panel). Dimension 2 combined histological features that highlighted the differences between other perinatal animals and adults with an important contribution of glycogen storage, which was biased toward category G2 (diffuse glycogen storage) in perinatal controls and cloned calves. Although fibrosis (F2) and inflammation (I1I2) were more frequently observed in adult clones than in adult AI, there was no significant difference between these two groups. These results therefore demonstrate that in the present cohort, all clones that died before birth suffered from severe histological lesions of the liver.

These findings caused us to determine quantitative measurements of these hepatic injuries by means of a morphometric analysis. The nuclear area tended to be smaller in perinatal clones (Fig. 1C), and the nuclear shape was also altered, as revealed by the reduced shape factor. Consistent with the anisocytosis observed in Fig. 1A,B, the nuclei of perinatal clones displayed greater heterogeneity in size and shape than those of perinatal AI controls (Fig. 1D; increased coefficients of variation (CVs)). Perinatal clones tended to have smaller hepatocytes (p = 0.1145, Fig. 1E, left panel) with a more irregular shape (p = 0.0520, right panel) than AI controls. The tendency towards smaller hepatocytes was more marked in cloned foetuses (which failed to accumulate glycogen) than in cloned calves, so we tested whether the cell size differed in individuals with (G2) and without (G0) glycogen storage. The cell area was indeed significantly larger in the G2 group than in the G0 group which contained most cloned foetuses (median G2 group = 247 μm^2^; median G0 group = 153 μm^2^; p = 0.0007), showing that sizing the hepatocytes provided an indirect measure of the level of glycogen storage. No significant differences could be found between adult clones and adult controls (Supplementary Fig. S1). Taken together, these data demonstrate that quantitative measures reflecting key histopathological features of perinatal clones could be obtained by means of a morphometric analysis of hepatocytes.

### The fatty acid composition is altered in perinatal clones

**Figure 2:**
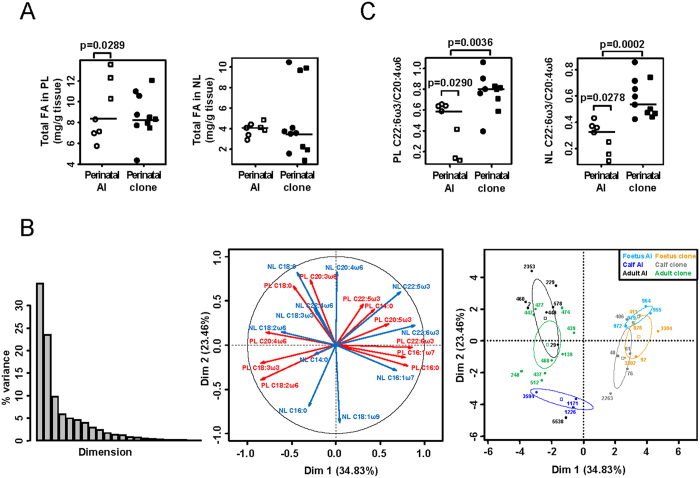
Altered FA composition in the livers of pathological perinatal clones. (**A**) The total amount of FAs was measured by gas chromatography in phospholipids and neutral lipids and expressed as mg/g liver. Each dot represents one animal. Open dots: perinatal AI controls; filled dots: perinatal clones. Prenatal (circles) and postnatal (squares) animals were considered separately. Significant differences between prenatal and postnatal animals are indicated (p < 0.05, permutation test). PL; phospholipids; NL: neutral lipids. (**B**) For 22 FAs each measured on 34 individuals and detected in both adults and perinatal animals, PCA was run on FA percentages converted to rankings in order to homogenize the distributions. Left panel: barplot of the contribution of each dimension to total variance; middle panel; variable factor map with FAs belonging to phospholipids and neutral lipids shown in red and blue, respectively; right panel: individual factor map with 95% confidence ellipses shown for each group. (**C**) The ratio between C22:6ω3 and C20:4ω6 was determined in the phospholipids and neutral lipids of each animal. Significant differences between perinatal AI and perinatal clones are indicated, as are significant differences between prenatal and postnatal animals (p < 0.05, permutation test).

To determine the molecular outcome of the hepatosteatosis observed in some cloned foetuses, we then addressed the issue of whether the quantity and composition of fatty acids (FA) were modified in clones. Twenty types of long-chain FAs were found in the perinatal animals, and 16 in the adults. Total FA levels of either membrane phospholipids or neutral lipids representing intracellular lipid storage, did not differ significantly between the perinatal clones and perinatal controls, although the elevated FA content in neutral lipids confirmed marked steatosis in three clones (Fig. 2A). Principal component analysis (PCA) run on the FA composition revealed that members of the omega 3 and omega 6 polyunsaturated FA families, such as docosahexaenoic acid (C22:6ω3) and arachidonic acid (C20:4ω6), were major contributors to dimensions 1 and 2 which explained 58% of total variance (Fig. 2B, left and middle panels). Dimension 1 essentially discriminated adults from perinatal clones and AI foetuses, while dimension 2 separated AI calves from the other animals (right panel). Cloned foetuses and cloned calves displayed an intermediary phenotype between AI foetuses and AI calves. However, it is worth noting that the two oldest cloned calves, individuals 76 and 2263 (postnatal days 2 and 4), tended to be more similar to the AI calves (postnatal day 4). Because the PCA highlighted C22:6ω3 and C20:4ω6 as potent FAs to partition the individuals according to age and clone status, we next focused on the C22:6ω3/C20:4ω6 ratio, which was significantly elevated in perinatal clones, independently of the pre/postnatal stage, with respect to both phospholipids and neutral lipids (Fig. 2C). In addition to the C22:6ω3/C20:4ω6 ratio, several major differences were found between perinatal AI and perinatal clones in terms of FA composition (Supplementary Fig. S2). Minor differences were also detected between adult clones and controls (Supplementary Fig. S3). Collectively, these data demonstrate that the FA composition was altered in the livers of the cloned animals. In perinatal clones, these alterations particularly affected the bioactive polyunsaturated FAs C22:6ω3 and C20:4ω6.

### Birth-induced liver maturation does not occur in pathological perinatal clones

**Figure 3:**
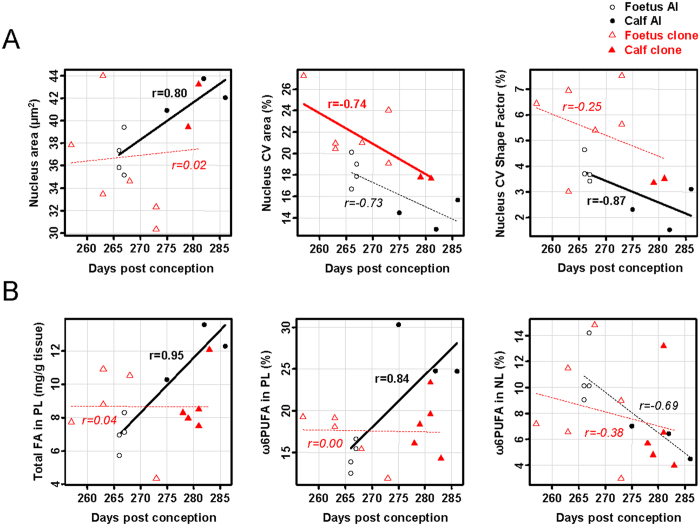
Altered biological age of the liver in pathological perinatal clones. Phenotypic measurements on hepatocyte nuclei (**A**) or FA levels and composition (**B**) are plotted against chronological age, assessed as the interval in days between conception (AI for controls, nuclear transfer for clones) and death/necropsy. This measurement of chronological age allows a representation of prenatal animals (open dots) and postnatal animals (filled dots) on the same graph, independently of the timing of birth. The black circles and red triangles indicate perinatal AI controls and perinatal clones, respectively. The least squares lines of best fit and Spearman’s rank correlation coefficients r are shown in black and red for perinatal AI controls and perinatal clones, respectively. Significant correlations (Spearman’s rank correlation test; p < 0.05) are indicated by plain lines and correlation coefficients in bold letters, whereas non-significant correlations (p ≥ 0.05) are indicated by dotted lines and correlation coefficients in italics. PL: phospholipids; NL: neutral lipids; ω6PUFA: omega 6 polyunsaturated FAs.

To investigate whether physiological maturation of the liver at birth had effects on the phenotypic parameters measured, we compared foetuses with calves in both the AI and clone groups. For most histomorphometric parameters and FA features, we observed significant differences between the prenatal and postnatal stages in AI controls only (Figs 1C,D and 2A,C and Supplementary Fig. S2). The absence of physiological changes in perinatal clones probably did not result solely from an uneven age distribution (i.e., most cloned calves analysed here were at pseudo-term whereas the AI calves were at postnatal day 4). Indeed, the correlation between chronological age and phenotype was lost in perinatal clones regarding the morphometric parameters of hepatocyte nuclei (except for the nucleus CV area; Fig. 3A and Supplementary Fig. S4). FA levels and composition also tended to correlate to chronological age in perinatal AI, with a correlation coefficient of r ≥ 0.5 for most of the individual FAs examined (26 out of 34; Supplementary Fig. S4). By contrast, in perinatal clones, only six individual FAs tended to correlate to chronological age. This tendency was exemplified by the total amounts of FAs and the percentage of omega 6 polyunsaturated FAs in phospholipids, both positively correlated to chronological age, and the percentage of omega 6 polyunsaturated FAs in neutral lipids, which tended to correlate negatively to chronological age, all correlations being observed in AI controls only (Fig. 3B). We concluded that birth-induced maturation did not occur and therefore that the biological age of the liver was altered in the pathological perinatal clones.

### Two distinct sets of differentially methylated regions underlie the normal transition to adult life and abnormal liver physiology

The next objective was to investigate whether a correlation could be established between the phenotypic modifications described above and altered DNA methylation profiles. We identified differentially methylated regions (DMRs) in gene promoters displaying variations between perinatal animals and adults (246 age-related DMRs) and/or between clones and AI controls (83 cloning-related DMRs) (Supplementary Information). Compared to the perinatal controls, adults were globally demethylated at age-related DMRs, indicating that dynamic changes in DNA methylation paralleled the normal transition from perinatal to adult life (Supplementary Fig. S12). Similarly, pathological perinatal clones displayed aberrant hypomethylation at most age-related DMRs and cloning-related DMRs, suggesting epigenetic alterations underlying an abnormal liver physiology. To highlight DMRs associated with phenotypic variations, we used multiple factor analysis (MFA)^33^. Three sets of quantitative variables were integrated: methylation at age-related DMRs and cloning-related DMRs (“DMR” set, 282 variables), histomorphometric parameters measured on hepatocytes (“Morpho” set) and FA composition (“FA” set).

**Figure 4:**
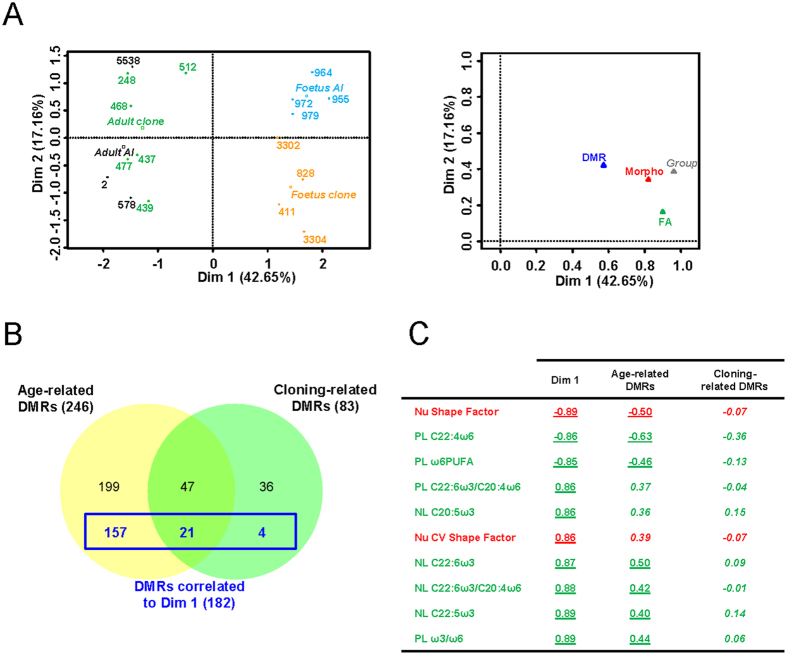
Age-related DMRs underlie the normal transition to adult life. (**A**) MFA was run on a subset of animals using three sets of quantitative variables: DMR (methylation at age-related DMRs and cloning-related DMRs, 282 variables; Supplementary Information), Morpho (histomorphometric parameters; 4 variables) and FA (FA features in phospholipids and neutral lipids; 48 variables). The group (adult AI, adult clone, foetus AI and foetus clone) was set as the illustrative variable. Left panel: graphic display of individuals in the principal plane of MFA (mean representation of individuals according to the three datasets). Right panel: representation of each set of variables including the illustrative variable in the principal plane of MFA, with contributions to dimensions 1 and 2 given by the coordinates of their projections on the x-axis and y-axis, respectively. (**B**) 182 variables of the DMR set were significantly correlated to dimension 1 of MFA. The graph indicates the distribution of these variables (in blue) among age-related DMRs and cloning-related DMRs. (**C**) Variables of the Morpho set (in red) and FA set (in green) that were the most highly significantly correlated to dimension 1 of MFA are listed. Correlation coefficients with dimension 1 of MFA (n = 17 individuals; correlation coefficients produced as an output of MFA) and with average DNA methylation at age-related DMRs and cloning-related DMRs (n = 19 to 25; Spearman’s rank correlation coefficients) are given in the Dim 1, Age-related DMRs and Cloning-related DMRs columns. For age-related DMRs and cloning-related DMRs, the variables tested for correlations with phenotypic variables are Pi_age_ and Pi_cloning_ for individual i, respectively (see Methods). For a significant correlation (p < 0.05) the correlation coefficient is underlined, and is otherwise displayed in italics. Nu: histomorphometric measurements on hepatocyte nuclei; PL: phospholipid fraction; NL: neutral lipid fraction; ω3/ω6: ratio between omega 3 and omega 6 polyunsaturated FAs.

First of all, MFA was applied to the largest set of individuals with no missing data, which included adult controls, adult clones, AI foetuses and cloned foetuses. The first dimension of MFA combined variables from the different sets that provided a clear partition between adults and foetuses (Fig. 4A, left panel). In the same way as for the FA and Morpho sets, the coordinate of the “Group” illustrative variable on dimension 1 was close to 1, while its coordinate on dimension 2 was quite weak. This showed that partitioning as a function of groups was particularly relevant on dimension 1, and that the phenotypic datasets made a major contribution to this (right panel). Variables in the three sets contributing to this partitioning could be identified based on their strong correlation with dimension 1. Interestingly, the 182 DMRs significantly correlated to dimension 1 (listed in Supplementary Table S6) were almost exclusively limited to age-related DMRs (Fig. 4B). The phenotypic variables most significantly correlated to dimension 1 are shown Fig. 4C and notably included modifications to the percentages of omega 3 and omega 6 polyunsaturated FAs. Consistent with the observation that only age-related DMRs contributed to dimension 1 of MFA, significant correlations were observed between these phenotypic variables and the average methylation at age-related DMRs, but not at cloning-related DMRs (Fig. 4C). Gene ontology (GO) analysis of the 182 DMRs that correlated to dimension 1 of MFA revealed an over-representation of genes involved in glycogen, lipid and cholesterol metabolism (Supplementary Fig. S14). This suggests that modifications to DNA methylation profiles regulate the expression of genes involved in the major metabolic pathways of the liver during the switch towards an adult diet. Most of these genes were methylated in perinatal animals and demethylated in adults, without there being any significant effect of cloning. Overall, these results show that when all the datasets were considered, the major source of variability that could be identified was related to age and not to cloning. They also demonstrate that a substantial part of the age-related DMRs correlated to the phenotypic variations that distinguished perinatal from adult animals.

**Figure 5:**
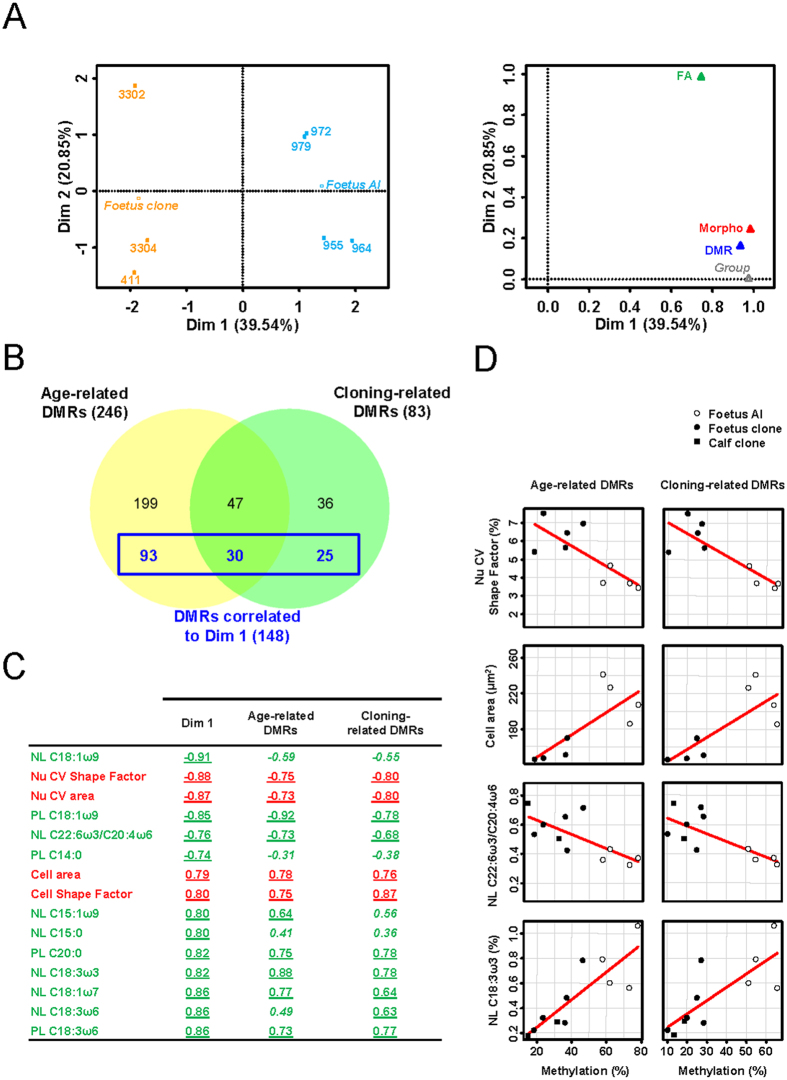
A subset of age-related DMRs and cloning-related DMRs underlie abnormal liver physiology. (**A**) MFA was run on seven foetuses using three sets of quantitative variables: DMR (methylation at age-related DMRs and cloning-related DMRs, 282 variables), Morpho (histomorphometric parameters, 8 variables) and FA (FA features, 56 variables). The group (foetus AI and foetus clone) was set as the illustrative variable. Left panel: graphic display of individuals in the principal plane of MFA (mean representation of individuals according to the three datasets). Right panel: representation of each set of variables including the illustrative variable in the principal plane of MFA. (**B**) Variables of the DMR set significantly correlated to dimension 1 of MFA (in blue) were evenly distributed among age-related DMRs and cloning-related DMRs. (**C**) Variables of the Morpho set (in red) and FA set (in green) that were significantly correlated to dimension 1 of MFA are listed. Correlation coefficients with dimension 1 of MFA (n = 7 individuals; correlation coefficients produced as an output of MFA) and with average DNA methylation at age-related DMRs and cloning-related DMRs (n = 8 to 11; Spearman’s rank correlation coefficients) are given in the Dim 1, Age-related DMRs and Cloning-related DMRs columns. For a significant correlation (p < 0.05) the correlation coefficient is underlined, and is otherwise displayed in italics. Nu: histomorphometric measurements on hepatocyte nuclei; Cell: histomorphometric measurements on hepatocytes; PL: phospholipids; NL: neutral lipids. (**D**) Significant correlations between four phenotypic variables shown in (**C**) and average DNA methylation at age-related DMRs and cloning-related DMRs. Each dot corresponds to one animal. The x-axis represents the average percentage of enriched probes Pi calculated for each animal i at DMRs (left panel: Pi_age_; right panel: Pi_cloning_).

Dimension 2 of MFA tended to separate AI foetuses from cloned foetuses while adult clones and adult controls were not dissociable, suggesting that the second important source of variability was related to the effects of cloning in foetuses only. To identify the variables that discriminated cloned and AI foetuses, we next ran MFA on a restricted subset of foetuses in which a more detailed phenotypic characterization had been performed and the number of variables had been increased (three clones and four controls). Dimension 1 combined variables in the three sets that discriminated AI from clones, highlighting a cloned/AI origin as the principal source of variability between foetuses from both the phenotypic and epigenetic points of view (Fig. 5A, left panel). The coordinate of the Group illustrative variable on dimension 1 was very close to 1, while its coordinate on dimension 2 was null, demonstrating that only dimension 1 enabled partitioning as a function of the cloned/AI origin (Fig. 5A, right panel). As illustrated by their elevated coordinates on dimension 1, both the phenotypic and DMR sets made a major contribution to this partitioning, while the FA set contributed equally to both dimensions 1 and 2. Dimension 2 therefore highlighted differences in FA composition in some individuals, independently of their cloned/AI origin. Unlike the DMRs contributing to the separation according to age, the 148 DMRs that significantly correlated to dimension 1 (listed in Supplementary Table S7) were evenly distributed among both age-related DMRs and cloning-related DMRs (Fig. 5B). The phenotypic variables that were significantly correlated to dimension 1 reflected heterogeneity in the morphology of hepatocyte nuclei (CVs), glycogen storage (cell area), the shape of hepatocytes (cell shape factor), the balance between omega 3 and omega 6 polyunsaturated FAs, and other FA features (Fig. 5C). Consistent with the MFA results, significant correlations with average methylation at both age-related DMRs and cloning-related DMRs were confirmed for most of these phenotypic variables in a larger set of animals (eight to eleven animals, Fig. 5D). Taken together, these results highlighted the phenotypic parameters that could distinguish pathological perinatal clones from perinatal AI controls and might therefore constitute markers of perinatal pathologies. They also demonstrated that a subset of both age-related DMRs and cloning-related DMRs were correlated to these markers.

### Genes involved in the response to stress and energy metabolism are deregulated in pathological perinatal clones

**Figure 6:**
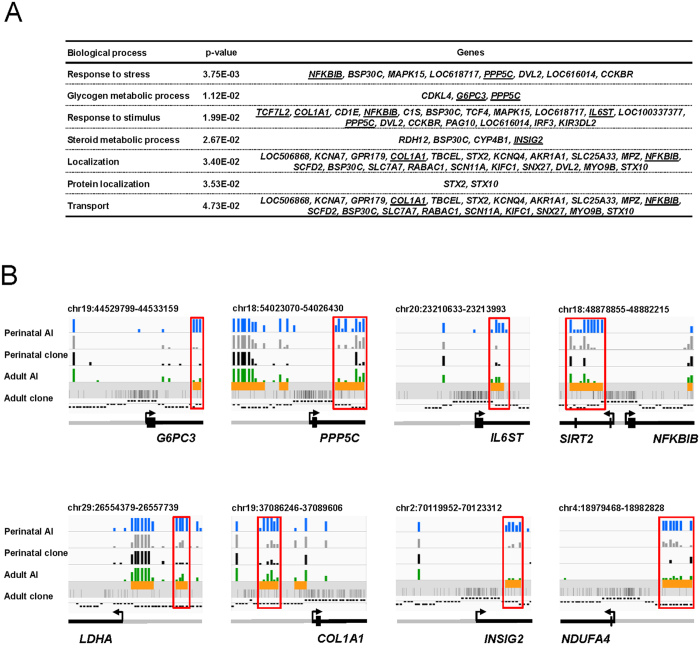
DMRs associated with abnormal liver physiology target genes involved in the response to stress and energy metabolism. (**A**) GO analysis performed using PANTHER on the 148 DMRs significantly correlated to dimension 1 of MFA and contributing to the separation between cloned and AI foetuses. Terms of biological processes displaying a statistical over-representation among the DMRs are indicated, as are the corresponding genes. Genes shown in B and in Fig. 7A are underlined. (**B**) IGV browser views showing regions targeted by the microarray for genes involved in energy metabolism (*G6PC3, PPP5C, LDHA, SIRT2, INSIG2* and *NDUFA4*), fibrosis (*COL1A1*) and inflammation (*IL6ST* and *NFKBIB*). The blue, grey, black and green bar charts represent the proportions of enriched probes at each probe position for the four groups of animals. Orange boxes indicate the regions of interest selected for differential analysis as defined in the Supplementary Methods. CpGs are shown, as are the probe classes based on CpG frequency (the upper, middle and lower bands represent high, intermediate and low class probes, respectively). DMRs are indicated by red boxes.

**Figure 7:**
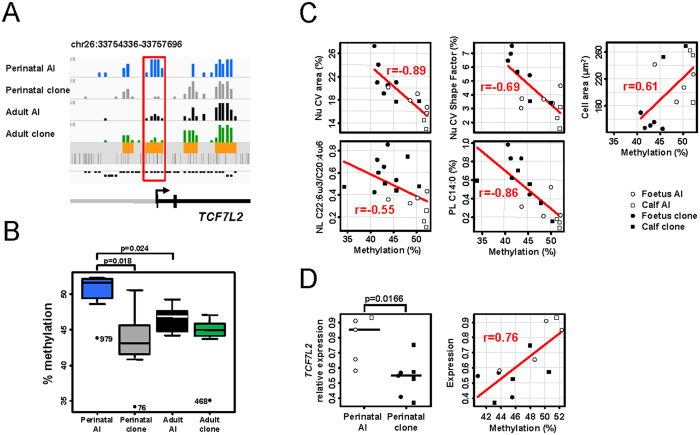
*TCF7L2* methylation and expression are deregulated in pathological perinatal clones. (**A**) IGV browser view showing the region targeted by the microarray for *TCF7L2*. The blue, grey, black and green bar charts represent the proportions of enriched probes at each probe position for the four groups of animals. The DMR is indicated by a red box. (**B**) Average methylation level of six CpGs assayed by pyrosequencing for perinatal AI (n = 7), perinatal clones (n = 11), adult AI (n = 8) and adult clones (n = 9). For each box, the middle line indicates the median and the edges the 25^th^/75^th^ percentiles. Significant differences between groups are indicated (p < 0.05, permutation test for pairwise comparisons). (**C**) Significant correlations between five phenotypic variables described in Fig. 5C and DNA methylation at *TCF7L2* DMR are shown (p < 0.05; Spearman’s rank correlation test). Each dot corresponds to one animal. The least squares lines of best fit and correlation coefficients are indicated. The x-axis represents the average percentage of methylation of six CpGs belonging to the *TCF7L2* DMR and obtained by pyrosequencing. Cell area is used as an indirect measure of the glycogen content of hepatocytes. Nu: histomorphometric measurements on hepatocyte nuclei; Cell: histomorphometric measurements on hepatocytes; PL: phospholipids; NL: neutral lipids. (**D**) The expression of *TCF7L2* was assessed by quantitative PCR. Left panel: the relative expression of *TCF7L2* was reduced in perinatal clones compared to perinatal controls (p < 0.05, permutation test). Right panel: positive correlation between DNA methylation assessed by pyrosequencing and expression (p < 0.05; Spearman’s rank correlation test).

GO analysis of the 148 DMRs that significantly correlated to dimension 1 of MFA and contributed to the separation between perinatal clones and controls revealed an over-representation of genes involved in the response to stress and stimuli (*NFKBIB, PPP5C, TCF7L2, COL1A1, IL6ST*) and in metabolic pathways (glycogen and steroids; Fig. 6A). As well as *TCF7L2, G6PC3, PPP5C* and *INSIG2* listed in Fig. 6A, other key genes in energy metabolism such as *LDHA, SIRT2* and *NDUFA4* were epigenetically deregulated in perinatal clones. The microarray data showed that these genes were hypomethylated in perinatal clones when compared to perinatal controls (Fig. 6B and Fig. 7A).

The identification of *TCF7L2* among the DMRs correlated to dimension 1 of MFA was appealing, because this is a master gene for liver differentiation and function^34^,^35^. Furthermore, the DMR was analysed by pyrosequencing on an extended cohort of animals (Fig. 7B), enabling a more detailed characterization of the relationships between methylation and phenotype. Among the phenotypic variables listed in Fig. 5C, five correlated significantly to methylation at *TCF7L2* DMR (Fig. 7C). Consistent with the function of *TCF7L2* in the regulation of gluconeogenesis, the livers of cloned foetuses that failed to store glycogen (reduced cell area) displayed a weaker methylation of *TCF7L2* than those of cloned calves or controls with normal glycogen storage. Furthermore, *TCF7L2* expression was weaker in the livers of perinatal clones than in those of perinatal controls, and was positively correlated to methylation of the DMR (Fig. 7D). The gene start of bovine *TCF7L2,* as defined at the time of the microarray design and in the current versions of bovine genome annotation, maps on coding exons in other species. It is therefore likely that *TCF7L2* DMR is centred on a coding exon rather than on the promoter, which is consistent with a positive correlation between methylation and expression^36^. In conclusion, the methylation status of genes critical to achieving a response to stress and to regulating energy metabolism was correlated to altered phenotypic parameters in pathological perinatal clones. For at least one of these genes, methylation defects were associated with deregulated expression, which may have functional effects on the liver phenotype in terms of response to stress and energy metabolism.

## Discussion

During this study, we explored the transition between late *in utero* life, early postnatal life and adulthood in terms of functional and epigenetic adaptations of the liver, in both conventional cattle and clones. Using multi-parameter correlation analyses, we demonstrated that normal physiological modifications associated with age, and phenotypic alterations related to perinatal mortality, were both underlain by specific changes to DNA methylation patterns.

In agreement with previous studies^28^,^29^, we observed important histological lesions in pathological cloned foetuses which included steatosis and severe fibrosis. In addition, we found that the fibrotic livers contained pseudo-canals of small hepatocytes reminiscent of regenerative nodules in cirrhotic livers. Unlike the foetal clones, the adult clones and cloned calves were not dissociable from age-matching AI controls in an analysis performed on scored lesions, suggesting that the liver damage affecting perinatal clones might be milder if gestation could be maintained until term. However, major alterations to FA composition and an absence of birth-induced maturation were indicative of hepatic defects in cloned calves as well. Modifications to quantitative histological parameters, such as the nuclear alterations observed in pathological perinatal clones, offered further evidence that they suffered from abnormal liver physiology and metabolism. Indeed, the size and shape of the interphase nucleus are closely dependent on cell size, function and metabolism and can be modified by ageing, oxidative stress and pathology^37^,^38^,^39^. Steatosis may also have contributed to the nuclear alterations we observed, since lipid vesicles compress the nucleus at the periphery of the hepatocyte, leading to its deformation. A Japanese survey of the necropsy findings relative to clones at various postnatal stages reported hepatic lesions in 14 out of 38 neonatal deaths, whereas this number dropped to two out of 25 at later stages of life^27^. This survey, together with the present study, both support the idea that pathological clones are characterized by an improper reprogramming of liver functions, which impairs their ability to adapt to postnatal life. We found epigenetic alterations correlated to phenotypic anomalies in genes related to the mitochondrial function (*NDUFA4, LDHA* and *SIRT2*), pointing towards mitochondrial defects as a potential origin of the liver damage seen in the pathological perinatal clones. In the context of chronic hepatitis C and non-alcoholic fatty liver disease (NAFLD), mitochondrial defects, together with inflammation and oxidative stress, are indeed suspected of causing the progression of hepatosteatosis towards more severe liver injury^40^,^41^. Interestingly, the hypothesis of mitochondrial defects in clones has been supported by other studies^42^. Variations in the proteomic profile of liver mitochondria have been observed in deceased newborn bovine clones^43^ and an altered expression of genes involved in oxidative phosphorylation, including *NDUFA4,* has been reported in the lungs of deceased cloned piglets^44^. *LDHA* is involved in the final step of glycolysis and is up-regulated in senescent cells with mitochondrial dysfunction^45^, and *SIRT2* is a key gene controlling entry into the pentose phosphate pathway^46^. The metabolic transition from oxidative phosphorylation to the glycolysis/pentose phosphate pathway favours NADPH-dependent antioxidant responses over energy expenditure, and occurs in the event of impaired mitochondrial function, ageing and cancer^47^. A genetic deficiency leading to an unbalanced metabolite flux in the pentose phosphate pathway results in an accumulation of reactive oxygen species, steatosis and fibrosis/cirrhosis in the livers of mice, and to *hydrops fetalis* and liver cirrhosis in human infants^48^. It is therefore possible that the similar symptoms induced by cloning may be caused by inadequate energy partitioning between the pentose phosphate pathway and oxidative metabolism.

Through its storage, export and β-oxidation of lipids, and lipogenesis, the liver plays a central role in lipid and FA metabolism in both pre-ruminant and ruminant cattle^49^, and several studies have reported changes to this metabolism in bovine clones^23^,^50^,^51^,^52^. At the blastocyst stage, different lipid metabolites were detected in the culture medium of two types of cloned embryos with contrasting term development abilities^50^. A transcriptomic analysis of cloned elongated conceptus highlighted the deregulation of genes involved in lipid and FA metabolism^51^, and in adult clones, both milk and meat displayed slight differences in their FA composition when compared to controls bred in the same facility^23^,^52^. The present study confirmed the small deviation in FA metabolism in an independent cohort of adult clones, and in another tissue, the liver. It also revealed important changes to FA metabolism in pathological perinatal clones, with an elevation of the C22:6ω3/C20:4ω6 ratio (which is related to the balance between anti- and pro-inflammatory FA derivatives) in both membrane and intracellular lipid stores. Given the lesions observed in the pathological perinatal clones, this result was surprising, since many studies have reported the beneficial actions of dietary omega 3 polyunsaturated FAs, including C22:6ω3, on hepatosteatosis and fibrosis (reviewed in ref. 53). The elevated C22:6ω3/C20:4ω6 ratio observed in pathological perinatal clones may have reflected a response to the hepatic injuries by boosting the pool of anti-inflammatory precursors such as C22:6ω3. Otherwise, it may have indicated the effects of a higher C20:4ω6 consumption in order to generate pro-inflammatory metabolites contributing to disease. During this study, we identified two potential candidates with functions in polyunsaturated FA metabolism that displayed epigenetic alterations in pathological perinatal clones: *ALOX15*, which metabolises C20:4ω6 to promote liver damage in hypercholesterolaemic mice^54^, and *CYB5R2 (LOC536960* in Supplementary Tables S4 and S7), which is involved in the desaturation and elongation of FAs and is a target of myristoylation^55^. Interestingly, myristic acid (C14:0) levels were raised in the membranes of our perinatal clones, and the protein encoded by *SIRT2* (which was differentially methylated in the perinatal clones) has been reported as displaying potent demyristoylase activity^56^. The regulation of *CYB5R2* at the gene level by methylation, and at the post-translational level by myristoylation, may therefore participate in the peculiar metabolism of omega 3 polyunsaturated FAs in pathological perinatal clones.

Hepatosteatosis and an absence of glycogen storage in cloned foetuses suggested a switch of intracellular energy stores from carbohydrates to lipids. We found aberrant DNA methylation profiles in two major effectors of the Wnt signalling pathway (*DVL2* and *TCF7L2*) that may be related to the impaired carbohydrate metabolism in perinatal clones. The functions of Wnt signalling in regulating hepatic glucose metabolism have been reported elsewhere^57^, and in humans, genetic variants and aberrant methylation profiles of *TCF7L2* have been associated with the development of type 2 diabetes^58^,^59^, increased NAFLD and fibrosis^60^. In line with a report showing that *TCF7L2* is necessary for gluconeogenesis and glycogen accumulation in the livers of neonates^34^, we demonstrated that *TCF7L2* methylation was positively correlated to both *TCF7L2* expression and hepatic glycogen storage in the perinatal clones. By contrast, others have shown that *TCF7L2* is a negative regulator of gluconeogenesis in adults^61^. These antagonistic roles may be a consequence of the complex transcriptional network in which *TCF7L2* is involved, with both transcriptional repressor and activator functions of its product depending on the interacting partners^62^. Because of this central position in the hepatic transcriptional network, *TCF7L2* is a master gene of liver phenotype, not only for glucose metabolism but also for that of lipids and amino acids^35^. The altered expression we observed in perinatal clones may therefore drive major pathophysiological changes in the liver, leading to NAFLD-related symptoms. In the perinatal clones, we also found epigenetic alterations in *G6PC3,* a target of *TCF7L2*^35^,^62^ which encodes a rate-limiting enzyme in gluconeogenesis. Together with other gluconeogenic genes, *G6PC3* is induced near term in response to the glucocorticoid surge, allowing glycogen to accumulate in order to supply energy to the newborn calf during the first hours post-partum^31^. This process failed to occur in our pathological cloned foetuses, but was at least partially corrected in the pathological cloned calves that were treated with dexamethasone before caesarean section (C-section). Dexamethasone, in combination with pre- or postnatal ACTH administration, is able to restore glycogen storage in the livers of premature calves^63^. It is therefore possible that in response to exogenous dexamethasone, the cloned calves initiated endogenous glucose production independently of the epigenetic deregulation of *G6PC3*.

The absence of glycogen storage, and the altered correlation between chronological age and the phenotype of the liver, indicated impaired liver maturation in the pathological perinatal clones. We demonstrated that as the normal transition to postnatal life occurred, hepatocytes became larger and acquired more regular and larger nuclei; the whole parenchyma matured towards a more homogeneous organization, and the membranes were enriched in FAs (and particularly in omega 6 polyunsaturated FAs that in parallel were depleted from intracellular FA stores). In pathological perinatal clones, all these processes were affected to different degrees, showing that the administration of dexamethasone was not sufficient to prevent a failure to mature towards a functional liver. In particular, the absence of maturation in terms of FA composition might have important consequences on membrane fluidity^64^ and on the FA composition of triglycerides exported by the liver. Cloned calves displayed normal cortisol secretion in response to ACTH^65^, suggesting that corticoid signalling, rather that corticoid secretion, was altered in perinatal clones. Altered corticoid signalling in perinatal clones may be related to the epigenetic deregulation of *PPP5C,* which antagonizes the anti-lipogenic actions of glucocorticoid receptor^66^. One can speculate that deregulated levels of *PPP5C* in the livers of cloned foetuses might impair the activation of anti-lipogenic genes in response to the glucocorticoid surge, leading to the derivation of gluconeogenic substrates toward fat accumulation. The inability of the immature liver to produce endogenous glucose may result in an increased dependence on maternal glucose, a hypothesis supported by reduced maternal plasma glucose levels and an elevated expression of placental glucose transporters in late cloned pregnancies^67^.

To our knowledge, this study is the first demonstration of an association between DNA methylation and liver phenotypic variations in cattle at a genome-wide scale. We showed that epigenetic alterations affected the promoters of genes relevant to hepatic metabolism in pathological perinatal clones. Using multi-parameter correlation analyses, we also revealed an association between these epigenetic alterations and phenotypic defects related to perinatal mortality. We did not observe any major physiological and epigenetic abnormalities in the livers of adult clones arising from the same donor cells as the perinatal clones, ruling out the effects of genotype on the phenotype observed. Our data therefore highlight epigenetics as a driving force behind phenotypic variability in farmed animals, and support the view that better control of the conditions of development is required in order to reduce perinatal losses and improve the long-term health of livestock species.

## Methods

### Animals and the collection of liver samples

All the methods described here were implemented in accordance with EU guidelines and regulations (directive 2010/63/UE), and all the experimental protocols were approved by the INRA local Ethics Committee (COMETHEA, authorization number 12/160).

*Post-mortem* liver samples were obtained from 36 Holstein cattle: eleven perinatal clones of two distinct genotypes displaying pathological lesions, nine adult clones (mostly clinically normal) of four distinct genotypes including the same genotypes as the perinatal clones, seven healthy perinatal controls resulting from AI and nine healthy adult AI controls. The animals, and the experiments performed on the liver samples, are described in Table 1. Because postnatal life induces considerable maturation of the liver, for the phenotypic investigations we considered four groups in perinatal animals: foetus AI, foetus clones, calf AI and calf clones. Animals that were the most homogeneous in terms of age, breeding and production period were used for the genome-wide methylation analysis and constituted the so-called “microarray cohort” (Supplementary Information). The “extended cohort”, used for pyrosequencing and phenotypic characterization, was obtained by including more animals in the microarray cohort, essentially perinatal clones and AI calves. Livers were sampled during the necropsy examination and were fixed immediately in 10% neutral buffered formalin or snap-frozen in liquid nitrogen and stored at −80 °C until use. Due to sampling limitations, autolysis or RNA degradation, not all the animals could be used for all the analyses.

This cohort of clones represented a decade of cloning during which the same procedure was applied continuously. For both clinically normal adult clones and pathological perinatal clones, somatic cell nuclear transfer was performed as described elsewhere^12^,^65^, using ear skin fibroblasts from four adult Holstein females. Bovine ovaries were collected from a slaughterhouse and cumulus oocyte complexes were aspirated from follicles 2–7 mm in diameter. Recipient oocytes were matured *in vitro*, and the metaphase II and polar body chromatin were removed 20–22 h post-maturation. For embryo reconstitution, an isolated donor cell was inserted under the *zona pellucida* of the enucleated oocyte and fused by electrostimulation. Embryos were seeded with Vero cells and cultured in B2 medium (CCD) supplemented with 2.5% foetal calf serum (Life Technologies). By day 7, blastocysts had been transferred into synchronized recipient heifers (two blastocysts per heifer), and the pregnant recipients were monitored by ultrasound imaging as recommended by the International Embryo Transfer Society (www.iets.org/pdf/hasac-healthassessmentcare.pdf). From gestational days 257 to 273, recipients positively diagnosed with hydrallantois were slaughtered at the INRA experimental facilities or delivered by C-section whenever possible, and the foetuses were processed for necropsy. Gestations were pursued until pseudo-term (gestational days 276 to 282) in the remaining recipients that were negative for hydrallantois, which represented half of the total recipients still pregnant at gestational day 150. To induce foetal maturation, a single dose of dexamethasone (20 mg) was injected 24 h before the C-section, and neonatal care was ensured in compliance with the guidelines of the International Embryo Transfer Society. Clones that died spontaneously within 4 days of birth were processed for necropsy, alongside those slaughtered because of severe limb malformations. Clones with a detectable heartbeat at birth were considered to be calves even if they died shortly after. All the other clones used here grew to adulthood and had a normal reproductive history, although their ages at culling tended to be younger than the controls (median ages at culling: 5 years for clones and 6.5 years for AI controls).

Perinatal and adult AI controls were maintained at the same experimental farm as the clones (UCEA, INRA, France), and both clones and controls were fed the same diet after birth. Adults were essentially fed with fodder and silage, depending on the physiological lactation stage. The recipients and mothers of all perinatal animals belonging to the microarray cohort were fed exclusively with fodder. In an attempt to improve the condition of the recipients of clones and thereby increase the chances of obtaining full-term gestations, other perinatal animals (individuals 40, 61, 76, 92, 1171, 1226 and 3594) were exposed to a different diet during gestation (fodder plus silage).

### Histological evaluations

Samples were embedded in paraffin wax and 6 μm-thick sections were routinely stained with haemalin-eosin-saffron. For 30 animals, seven types of lesions or histological features were scored by a skilled pathologist (Tables 1 and 2). Morphometric measurements were performed using a digital camera (Nikon DXM 1200) combined with Nikon Imaging Software (Nikon). Microscopic fields were selected randomly on haemalin-eosin-saffron-stained sections using high magnification. Nuclear size and shape were used as indicators of the anisocytosis observed in some individuals. For at least 200 hepatocyte nuclei per animal on several separate fields, minimum and maximum Feret diameters, total area, perimeter and shape factor (ranging from 0 to 1, with 1 corresponding to perfect circles) were measured. As the size parameters were highly correlated, only the area and shape factor were considered. Furthermore, hepatocyte size and shape were used to determine available energy stores. For perinatal animals in which the cell membranes of hepatocytes were clearly observable, cell total area and shape factor were measured on 35 hepatocytes on several separate fields. For each animal and histomorphometric parameter, the mean and CV were calculated. AI controls and clones were compared using a permutation test for two independent samples (Monte-Carlo sampling of 100,000 permutations), which is a non-parametric test suited to small samples. For perinatal animals, a stratified permutation test was used to take account of the stage (pre/postnatal). Foetuses and calves were then compared independently within each group. Correlations related to morphometric measurements were estimated using Spearman’s rank correlation test.

### Fatty acid composition

Lipids corresponding to 100 mg of hepatic tissue were extracted using chloroform-methanol. Phospholipids were separated from non-phosphorous lipids on silica acid cartridges. The phospholipids and neutral lipids were transmethylated with 7% Boron trifluoride methanol (Sigma-Aldrich). The methyl esters of phospholipids or neutral FAs were analysed by gas chromatography coupled to FID (Gas Chromatograph 3900 Varian) on an Econo-Cap EC-WAX capillary column, using heptadecanoic acid (C17:0) as the internal standard^68^. The results were expressed as mg/g liver or as percentages of total FAs. FAs that were only detected as traces were not considered. Statistical analyses relative to FA composition were performed as explained for the histological evaluation.

### Epigenetic analyses

The detailed methods are described in the Supplementary Information. Briefly, a Roche-NimbleGen 3 × 720 K microarray targeting the upstream region (−2000 to +1360 bp relative to the gene start) of 21,296 bovine genes was designed. Half of these upstream regions contained a CpG island. DNA extraction from the liver samples, methylated DNA immunoprecipitation (MeDIP) and quality controls were performed as described elsewhere^69^. To prevent any technical biases, the products of 9–10 independent MeDIP experiments were pooled for each animal. After moderate genome amplification, the pooled MeDIP reactions and corresponding input DNA were labelled with Cy3 and Cy5 and hybridized on the microarray, with technical dye-swaps for every sample.

Probes with signal enrichment in the MeDIP sample (“enriched probes”) were identified and an anchor-extension strategy was used to delineate 15,885 clusters of probes enriched under at least one condition (regions of interest). 246 age-related DMRs and 83 cloning-related DMRs were identified using an R package designed to analyse point patterns, which was therefore appropriate for binary data (enriched *vs.* not enriched). Three models were built: one full model taking account of both age and cloning (i) and two alternative models taking account of age only (ii) or cloning only (iii). The interaction between age and cloning was not considered. Indeed, a more robust estimation of only two parameters was preferred, given the limited sample size (n = 26). To identify cloning-related DMRs and age-related DMRs, the full model was compared with alternative models (ii) and (iii), respectively. All 15,885 regions were tested and the resulting p-values were corrected for multiple testing. A given region was considered as a cloning-related DMR if the full model fitted the observations significantly better than alternative model (ii) (adjusted p-value < 0.05). Similarly, a given region was considered as an age-related DMR if the full model fitted the observations significantly better than alternative model (iii). The microarray results were validated by the pyrosequencing analysis of 12 DMRs (115 analysed CpGs) on the extended cohort (n = 35).

### Multivariate analyses

Some theoretical background on the multivariate analyses used (MCA, PCA and MFA) is provided in the Supplementary Methods. All multivariate analyses were computed using the FactoMineR R package.

MCA (which has proved its usefulness in the field of histopathology^32^) was run on the set of qualitative data generated by scoring seven types of lesions in 30 animals (Tables 1 and 2). To prevent any distortion of the dimensions due to poorly represented categories, those containing fewer than four individuals (G1, S1, S2, A3, and I2) were merged together or with adjacent categories, generating the intermediate categories G1G2, S1S2, A2A3, and I1I2.

PCA was computed on 22 quantitative variables representing percentages of individual FAs measured in 34 individuals and detected in both adults and perinatal animals (C14:0, C16:0, C16:1ω7, C18:0, C18:1ω9, C18:2ω6, C18:3ω3, C20:3ω6, C20:4ω6, C20:5ω3, C22:4ω6, C22:5ω3, C22:6ω3 in both phospholipids and neutral lipids). Rank-converted percentages were used to homogenize the distributions. In the results presented in Fig. 2B, the PL C18:1ω9, NL C20:3ω6, PL C22:4ω6 and NL C20:5ω3 variables, which were respectively highly correlated to NL C18:1ω9, NL C20:4ω6, PL C20:4ω6, NL C22:5ω3, were removed, but this did not substantially change the results.

MFA, which enables an estimation of the relationships between sets of variables measured in the same individuals without *a priori* modelling^33^, was run on three sets of quantitative variables: the “DMR” set, the “Morpho” set and the “FA” set. The DMR set included all age-related DMRs and cloning-related DMRs (282 in total); the variables considered were the percentage of enriched probes 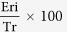, where Eri is the number of enriched probes for DMR r and individual i, and Tr is the number of probes included in DMR r. The variables included in the Morpho and FA sets were as follows: Fig. 4A shows the Morpho set containing four variables (mean area and mean shape factor measured on hepatocyte nuclei and the corresponding CVs) and the FA set including rank-converted percentages or ratios of FAs detected in both adult and perinatal animals: C14:0, C16:0, C16:1ω7, C18:0, C18:1ω9, C18:2ω6, C18:3ω3, C20:3ω6, C20:4ω6, C20:5ω3, C22:4ω6, C22:5ω3, C22:6ω3, total saturated FAs, total monounsaturated FAs, total polyunsaturated FAs, omega 6 polyunsaturated FAs, omega 3 polyunsaturated FAs, omega 3/omega 6, polyunsaturated FAs/saturated FAs, saturated FAs/unsaturated FAs, monounsaturated FAs/saturated FAs, C18:2ω6/C20:4ω6 and C22:6ω3/C20:4ω6 for both phospholipids and neutral lipids (48 variables). Additional variables were considered in Fig. 5A: mean cell area and mean cell shape factor measured on hepatocytes and the corresponding CVs (total of eight variables for the Morpho set), and PL 18:3ω6, PL 20:0, NL C14:1ω9, NL C15:0, NL C15:1ω9, NL C16:1ω9, NL C18:3ω6 and NLC18:1ω7 (total of 56 variables for the FA set). MFA was computed on these three datasets, with the animal group set as the illustrative variable. Individuals that constituted missing data for the considered variables were excluded from the analysis. In Figs 4C and 5C, correlations between the most contributory Morpho and FA variables and average values for methylation at age-related DMRs or cloning-related DMRs were confirmed using Spearman’s rank correlation test on an extended panel of animals. The variables considered for average methylation at age-related DMRs and cloning-related DMRs were Pi_age_ and Pi_cloning_, respectively, where 
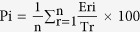
 and n is the total number of DMRs (n = 246 for age-related DMRs and n = 83 for cloning-related DMRs). GO analysis of the DMRs correlated to dimension 1 of MFA was performed using the PANTHER database^70^.

### Quantitative PCR

RNA was extracted with Trizol reagent (Invitrogen) according to the manufacturer’s instructions, and reverse transcription and real-time quantitative PCR were performed as described elsewhere^71^. GeNorm software^72^ was used to select *RPLP0, RPL19* and *YHAWZ* as reference genes for normalization and to obtain normalization factors. The primers for *RPLP0* and *RPL19* are described elsewhere^71^, as are the primers for *YHAWZ*^73^. The primers for *TCF7L2* were as follows (5′-3′): bTCF7L2_ex5_F1, AGCTGACGTAGACCCCAAAA; bTCF7L2_ex6_R1, TAGCGGATGGGGGATTTGTC. The results were presented as the mean relative expression calculated for the sample in four replicates. Statistical analyses were performed as described in the section on histological evaluation.

## Additional Information

**Accession codes:** The MeDIP-chip dataset supporting the results of this article is available in the NCBI Gene Expression Omnibus database (http://www.ncbi.nlm.nih. gov/geo/) under accession number GSE73028.

**How to cite this article**: Kiefer, H. *et al*. Altered DNA methylation associated with an abnormal liver phenotype in a cattle model with a high incidence of perinatal pathologies. *Sci. Rep.* **6**, 38869; doi: 10.1038/srep38869 (2016).

**Publisher's note:** Springer Nature remains neutral with regard to jurisdictional claims in published maps and institutional affiliations.

## Supplementary Material

Supplementary Information

Supplementary Dataset 1

Supplementary Dataset 2

Supplementary Dataset 3

Supplementary Dataset 4

## Figures and Tables

**Table 1:** Animals.

Animal	Breed/Genotype	Sex	Age	Cause of death/phenotype	Parity/ Lactations	Histological characterization	Morphometrical measurements	Fatty acid composition	Methylation assay
Foetus AI									
*955	Holstein	F	GD267	Slaughter of the mother/Normal	0/0	R0, F2, G2, S0, C0, A1, I0	Nucleus, Cell	PL, NL	Microarray, pyrosequencing
*964	Holstein	M	GD267	Slaughter of the mother/Normal	0/0	R0, F0, G2, S0, C0, A1, I0	Nucleus, Cell	PL, NL	Microarray, pyrosequencing
*972	Holstein	M	GD266	Slaughter of the mother/Normal	0/0	R0, F0, G2, S0, C0, A1, I0	Nucleus, Cell	PL, NL	Microarray, pyrosequencing
*979	Holstein	F	GD266	Slaughter of the mother/Normal	0/0	R0, F0, G2, S0, C0, A3, I0	Nucleus, Cell	PL, NL	Microarray, pyrosequencing
Foetus clone									
92^1^	Holstein/5538	F	GD263	Spontaneous death after C-section/Hydrops	0/0	R2, F3, G0, S0, C1, A0, I0	Nucleus, Cell	PL, NL	Pyrosequencing
*411	Holstein/2251	F	GD268	Spontaneous abortion/Hydronephros	0/0	R2, F3, G0, S1, C2, A1, I0	Nucleus, Cell	PL, NL	Microarray, pyrosequencing
*828	Holstein/2251	F	GD263	Slaughter of the recipient/Hydrops	0/0	R2, F3, G2, S0, C1, A2, I0	Nucleus	PL, NL	Microarray, pyrosequencing
*3302	Holstein/5538	F	GD257	Spontaneous abortion/Hydrops	0/0	R2, F3, G0, S1, C2, A2, I0	Nucleus, Cell	PL, NL	Microarray, pyrosequencing
*3303^2^	Holstein/5538	F	GD273	Stillborn at C-section/Hydrops	0/0	R2, F2, G0, S1, C0, A2, I0	Nucleus, Cell	NL	Microarray, pyrosequencing
*3304^2^	Holstein/5538	F	GD273	Stillborn at C-section/Hydrops	0/0	R2, F0, G0, S2, C2, A2, I0	Nucleus, Cell	PL, NL	Microarray, pyrosequencing
Calf AI									
1171	Holstein	F	PD4	Slaughter/Normal	0/0	R1, F0, G0, S2, C0, A1, I0	Nucleus, Cell	PL, NL	Pyrosequencing
1226	Holstein	F	PD4	Slaughter/Normal	0/0	R0, F0, G2, S0, C0, A0, I0	Nucleus, Cell	PL, NL	Pyrosequencing
3594^1^	Holstein	F	PD4	Slaughter/Free Martin	0/0	R0, F0, G2, S0, C0, A1, I0	Nucleus, Cell	PL, NL	Pyrosequencing
Calf clone									
40^1^	Holstein/5538	F	Term (GD279)	Slaughter 1 h after birth/Severe limb deformities	0/0	R0, F0, G2, S0, C0, A0, I0	Nucleus, Cell	PL, NL	Pyrosequencing
61	Holstein/5538	F	Term (GD281)	Spontaneous death 5 min. after birth/Cardiac failure	0/0	R0, F0, G2, S0, C0, A0, I0	Nucleus, Cell	PL, NL	Pyrosequencing
76	Holstein/5538	F	PD2	Spontaneous death/Kidney dysfunction?	0/0	*NA*	*NA*	PL, NL	Pyrosequencing
*406	Holstein/2251	F	Term (GD281)	Spontaneous death 6 h after birth/Internal haemorrhage	0/0	R1, F3, G2, S0, C2, A0, I0	*NA*	PL, NL	Microarray, pyrosequencing
*2263	Holstein/5538	F	PD4	Slaughter/Limb deformities	0/0	*NA*	*NA*	PL, NL	Microarray, pyrosequencing
Adult AI									
*2	Holstein	F	10 years	Slaughter/Normal	7/7	R0, F0, G0, S0, C0, A0, I1	Nucleus	PL, NL	Microarray, pyrosequencing
29	Holstein/0029^3^	F	10 years	Slaughter/Normal	1/1	R1, F0, G0, S0, C0, A0, I0	Nucleus	PL, NL	*NA*
*229	Holstein	F	8 years	Slaughter/Normal	5/5	*NA*	*NA*	PL, NL	Microarray, pyrosequencing
*428	Holstein	F	6 years	Slaughter/Normal	4/3	R0, F0, G0, S0, C0, A1, I0	Nucleus	*NA*	Microarray, pyrosequencing
*449	Holstein	F	6 years	Slaughter/Normal	2/2	R0, F0, G0, S0, C0, A0, I1	*NA*	PL, NL	Microarray, pyrosequencing
*460	Holstein	F	6 years	Slaughter/Normal	2/2	R1, F2, G0, S0, C0, A2, I1	*NA*	PL, NL	Microarray, pyrosequencing
*578	Holstein	F	5 years	Slaughter/Normal	1/1	R0, F0, G0, S0, C0, A0, I1	Nucleus	PL, NL	Microarray, pyrosequencing
*2353	Holstein	F	7 years	Slaughter/Normal	*NA*	*NA*	*NA*	PL, NL	Microarray, pyrosequencing
*5538	Holstein/5538^3^	F	15 years	Slaughter/Limb injury	7/6	R1, F2, G0, S0, C0, A1, I0	Nucleus	PL, NL	Microarray, pyrosequencing
Adult clone									
139	Holstein/7711	F	11 years	Slaughter/Normal	0/0	*NA*	*NA*	PL, NL	Pyrosequencing
*248	Holstein/5538	F	6 years	Slaughter/Limb infection	3/3	R0, F2, G0, S0, C0, A0, I2	Nucleus	PL, NL	Microarray, pyrosequencing
*437	Holstein/0029	F	6 years	Slaughter/Normal	2/2	R0, F2, G0, S0, C0, A0, I1	Nucleus	PL, NL	Microarray, pyrosequencing
*439	Holstein/2251	F	4 years	Slaughter/abnormal kidney	1/0	R0, F2, G0, S0, C0, A0, I0	Nucleus	PL, NL	Microarray, pyrosequencing
*447	Holstein/0029	F	5 years	Slaughter/Normal	2/2	R0, F0, G0, S0, C0, A0, I0	*NA*	PL, NL	Microarray, pyrosequencing
*468	Holstein/5538	F	5 years	Slaughter/Normal	2/2	R0, F2, G0, S0, C0, A1, I2	Nucleus	PL, NL	Microarray, pyrosequencing
474	Holstein/2251	F	8.5 years	Slaughter/Normal	4/3	*NA*	*NA*	PL, NL	Pyrosequencing
*477	Holstein/5538	F	3.5 years	Slaughter/Limb injury, abnormal kidney	1/1	R0, F2, G0, S0, C0, A0, I1	Nucleus	PL, NL	Microarray, pyrosequencing
*512	Holstein/5538	F	3 years	Slaughter/Limb injury	1/1	R0, F2, G2, S0, C0, A0, I1	Nucleus	PL, NL	Microarray, pyrosequencing

F: female; M: male; GD: gestational day; PD: postnatal day; *NA*: not assessed; PL: phospholipids; NL: neutral lipids. In the “Histological characterization” column, animals are graded according to the severity of their lesions (see Table 2). The following parameters were inspected: R: alteration of cell rows (Remak trabeculae); F: fibrosis; G: glycogen storage; S: steatosis; C: anisocytosis; A: aspect of sinusoidal capillaries and aneurysm; I: inflammation.

^*^Microarray cohort.

^1^Two foetuses developed but only one was included in the study.

^2^Same recipient cow.

^3^Cell donors for nuclear transfer (same genotype as some of the clones).

**Table 2:** Scoring of histopathological features.

Histopathological feature	Score	Description
Alteration of rows of hepatocytes (Remak trabeculae)	R0	No alteration
	R1	Only centrolobular trabeculae visible
	R2	No trabeculae
Fibrosis	F0	Absent
	F1	Bridging portal fibrosis
	F2	Dissecting fibrosis
	F3	Marked bridging fibrosis with nodules
Glycogen storage in hepatocytes	G0	Absent
	G1	Focal
	G2	Diffuse
Steatosis in hepatocytes	S0	Absent
	S1	Focal
	S2	Diffuse
Anisocytosis of hepatocytes	C0	Absent
	C1	Present
	C2	Present with some atypic cells
Aspect of sinusoidal capillaries	A0	Even diameter
	A1	Focal dilatation
	A2	Mild aneurysm
	A3	Important aneurysm
Inflammation	I0	Absent
	I1	Mild
	I2	Important
