# Supplementary material for: Altered DNA methylation associated with an abnormal liver phenotype in a cattle model with a high incidence of perinatal pathologies
Source: Sci Rep. 2016 Dec 13;6:38869. doi: 10.1038/srep38869 (PMC5153653; doi:10.1038/srep38869)
Supplement: Supplementary Information [file srep38869-s1.pdf]

Supplementary Information

Altered DNA methylation associated with an abnormal liver phenotype in a cattle model with a high incidence of perinatal pathologies

Hélène Kiefer[1*], Luc Jouneau[1], Évelyne Campion[1], Delphine Rousseau-Ralliard[1], Thibaut Larcher[2], Marie-Laure Martin-Magniette[3,4,5], Sandrine Balzergue[4,6], Mireille Ledevin[2], Audrey Prézelin[1], Pascale Chavatte-Palmer[1], Yvan Heyman[1], Christophe Richard[1,7], Daniel Le Bourhis[1,8], Jean-Paul Renard[1] and Hélène Jammes[1]

[1]UMR BDR, INRA, ENVA, Université Paris Saclay, 78350, Jouy en Josas, France

[2]INRA, UMR0703 APEX, Oniris, Nantes, France

[3]UMR MIA-Paris, AgroParisTech, INRA, Université Paris-Saclay, 75005, Paris, France

[4]Institute of Plant Sciences Paris Saclay IPS2, CNRS, INRA, Université Paris-Sud, Université Evry, Université Paris-Saclay, 91405 Orsay, France

[5]Institute of Plant Sciences Paris-Saclay IPS2, Paris Diderot, Sorbonne Paris-Cité, Bâtiment 630, 91405, Orsay, France

[6]Current address: IRHS, INRA, AGROCAMPUS-Ouest, Université d'Angers, SFR 4207 QUASAV, 42 rue Georges Morel, 49071 Beaucouzé cedex, France

[7]INRA, UE1298, Unité Commune d'Expérimentation Animale, Leudeville, France

[8]Current address: ALLICE, lieu-dit Le Perroi, Nouzilly, France

*Corresponding author

E-mail: Helene.Kiefer@inra.fr

Supplementary Results

Identification and characterization of differentially methylated regions in the bovine liver

To study the bovine DNA methylome at promoters we designed a tiling microarray targeting the upstream region of all annotated genes from -2000 to +1360 bp relative to the gene start. These regions are referred to as "promoters" hereinafter. In addition, potentially imprinted loci and surrounding regions, as well as one control region devoid of CpGs, were added to the microarray. These additional regions are referred to as "large regions" hereinafter (Supplementary Table S1). Genomic DNA was isolated from the liver of 26 Holstein cattle ("microarray cohort"): seven perinatal clones of two distinct genotypes with five of them being at late foetal stage, seven adult clones mostly clinically normal, of three distinct genotypes including the same as the perinatal clones, four healthy perinatal controls resulting from artificial insemination (AI) (including two males) and eight healthy adult AI controls (Table 1). This unique sampling, containing perinatal and adult cattle, males and females, pathological and healthy animals obtained using two reproductive technologies, provided an opportunity to describe the methylome of bovine liver under a broad range of conditions.

Methylated DNA was isolated by methylated DNA immunoprecipitation (MeDIP)[1] and hybridized on the microarray together with input DNA (Supplementary Fig. S5). Probes with signal enrichment in the MeDIP samples ("enriched probes" hereinafter) were identified using the ChIPmix program, which has been reported to outperform standard enrichment analysis based on the log ratio between IP and input[2]. Probes belonged to three classes according to their local percentage of CpGs, and ChIPmix was run independently for the three classes. Compared with an unique ChIPmix analysis where all probes are treated together and independent of their CpG content, this enabled an important gain in enriched probes in the low CpG class (<1 CpG per 100 bp; Supplementary Fig. S6) which is not efficiently processed using standard MeDIP analysis[3].

Consistent with the PCR quality controls performed prior to microarray hybridization (Supplementary Fig. S5), enriched probes could be identified in the large regions containing putative imprinted loci but not in unmethylated CpG-rich promoters such as *TBX15* or the *DAZL* negative control region devoid of CpGs (Supplementary Fig. S7). An additional control was provided by the relative number of enriched probes on the X chromosome, which differed between the two males and 24 females (Supplementary Fig. S8). This is probably related to X chromosome inactivation in females, which involves DNA methylation among other epigenetic mechanisms[4]. Except for the two males on the X chromosome, the relative number of enriched probes was remarkably conserved across individuals, independently of their age, sex or reproduction method. In addition, all autosomes displayed similar levels of enriched probes except for chromosome 18, which was highly methylated in all individuals. Interestingly, the local methylation of chromosome 18 was restricted to the portion sharing synteny with human chromosome 19, which has been reported to be highly methylated in circulating B cells[5].

As defined by the microarray design, promoters spanned an average of 32 probes. To determine whether enriched probes were clustered or not along the promoters, we performed a spatial analysis on all promoters with at least five enriched probes, using an R package designed to analyse point patterns[6]. This revealed that 96.7% of the analysed promoters displayed clusters of enriched probes, which might correspond to highly methylated regions. In order to focus our analysis on these regions of interest, we used an anchor-extension strategy to identify 15,885 clusters of probes enriched under at least one condition (mean size of these regions: 389 bp; average span: 4.5 probes). Most of these regions of interest (96.4%) were included in promoters, whereas 578 regions were located outside promoters and in the large regions. Approximately half of the 21,296 promoters present on the microarray contained at least one region of interest and could therefore be considered as methylated (Supplementary Fig. S9). Biological processes relevant to hepatic function, such as fatty acid biosynthesis, glycolysis and tricarboxylic acid cycle, the metabolism of cholesterol, steroids and amino acids, were over-represented among these methylated promoters.

A normalized factor NEpi, representing the number of enriched probes at promoter p for individual i, was computed for all promoters and individuals, and principal component analysis (PCA) was run on the resulting matrix (Supplementary Fig. S10A). Neither clone genotype, sex nor any other parameter examined so far could account for the segregation of individuals along dimensions 1 and 2, which together contributed to about 23% of total variance. However, clones and AI controls segregated as two different groups along dimension 3 (7.53% of total variance) and perinatal and adult animals were discriminated by dimension 5 (5.65% of total variance), suggesting that a small part of total variance was related to cloning and age, and that differentially methylated regions (DMRs) could be identified.

To determine DMRs displaying variations with age and cloning, we compared the proportion of enriched probes between perinatal and adult animals and between clones and AI controls, respectively, in each of the 15,885 regions of interest. A gradation in the severity of phenotypes could be observed in the present cohort of perinatal and adult clones: perinatal clones were all too pathological to support postnatal life, whereas adult clones had a reproductive and productive history quite similar to the controls (Table 1). Age has therefore been considered when comparing our set of clones with AI controls. Similarly, because the effect of cloning on development and ageing has not been completely elucidated, the reproductive technology has been considered when comparing perinatal animals with adults. Three models were built: one full model taking account of both age and cloning (i) and two alternative models taking account of age only (ii) or cloning only (iii). To identify cloning-related DMRs and age-related DMRs, the full model was compared with the alternative models (ii) and (iii), respectively. A given region of interest was considered as a DMR if the full model fitted the observations significantly better than the alternative model. This strategy led to the identification of 246 age-related DMRs and 83 cloning-related DMRs (Supplementary Tables S4, S5), the great majority of which (240 and 82) belonged to promoters. Strikingly, more than half of the cloning-related DMRs overlapped with age-related DMRs (Supplementary Fig. S10B), which was consistent with the tight correlation observed between the average percentages of enriched probes

per animal in age-related and cloning-related DMRs (Supplementary Fig. S11). Although the interaction between age and cloning has not been tested; the important extent of overlapping between age-related DMRs and cloning-related DMRs suggests intricate relationships between age and cloning in the determination of specific epigenetic profiles.

For each gene, the microarray targeted a 3.4 kb genomic window, which potentially included portions of intragenic regions in addition to gene upstream regions. We therefore aimed to localize the identified DMRs relative to certain functional elements present on the microarray (Supplementary Fig. S10C-D). We considered the most upstream ATG and exon definitions, and not the gene start which is not reliably annotated for all genes in cattle. The distributions of age-related DMRs and cloning-related DMRs displayed significant differences with the distribution of regions of interest. With 60.2% situated upstream of ATG and 83.7% outside exons, age-related DMRs seemed to be preferentially located in gene upstream regions. By contrast, cloning-related DMRs tended to be located within genes, since those found upstream of ATG were reduced to 43%. These distinct distributions suggested slightly different effects of DNA methylation on gene expression in the two sets of DMRs, because methylation at promoters is associated with gene repression while intragenic methylation seems to be necessary for proper splicing and gene expression[7].

Pathological perinatal clones display a demethylation inconsistent with their chronological age

In order to analyse the behaviour of DMRs, hierarchical clustering was performed on the number of enriched probes per DMR (Supplementary Fig. S12A-B). For age-related DMRs, the individuals segregated into two clearly distinct groups, one including all the perinatal AI controls and the other all the adult AI controls. This indicates that our strategy was successful in identifying relevant epigenetic signatures of age. Strikingly, clones were not distributed according to their age in these two groups, since each group contained both perinatal and adult clones. For cloning-related DMRs, hierarchical clustering separated clones and AI controls for perinatal animals only. Perinatal AI

controls were isolated from all other individuals, including perinatal clones that segregated with adults.

We next computed a global enrichment score (Pi) for each individual i (average percentage of enriched probes for the 246 age-related DMRs or 83 cloning-related DMRs) and compared the four groups using this variable (Supplementary Fig. S12C-D). For age-related DMRs, perinatal AI controls displayed the highest enrichment scores and were markedly hypermethylated compared to adult AI controls. By contrast, there was no significant difference between perinatal clones and adult clones. Perinatal clones were hypomethylated compared to perinatal AI controls, and adult clones were slightly but significantly more methylated than adult AI controls, which may be linked to the differences in age and production history between the two adult groups (median values for age, number of gestations/lactations: 5 years, 2/2 for clones and 6.5 years, 3/2.5 for AI controls). A global hypomethylation of perinatal clones was also observed in cloning-related DMRs (Supplementary Fig. S12D), where the enrichment scores of perinatal clones were closer to those of adults than to those of perinatal AI controls.

In order to further understand the effects of age and cloning on methylation at DMRs, we calculated the average percentage of enriched probes (Pr) at each DMR r in the four groups ($Pr_{perinatal\ AI}$, $Pr_{perinatal\ clones}$, $Pr_{adult\ AI}$, $Pr_{adult\ clones}$). We next plotted age-related DMRs according to the percentage of enriched probes in perinatal and adult animals, considering clones and AI controls separately. One DMR r is therefore represented by two dots, having the respective (x; y) coordinates ($Pr_{adult\ AI}$; $Pr_{perinatal\ AI}$) and ($Pr_{adult\ clones}$; $Pr_{perinatal\ clones}$). For AI controls, most of the DMRs (229 out of 246) were highly methylated in perinatal animals and demethylated in adults (Supplementary Fig. S12E), with a median $Pr_{perinatal\ AI}$-$Pr_{adult\ AI}$ difference of more than 50% enriched probes (Supplementary Fig. S12G). By contrast, the median $Pr_{perinatal\ clones}$-$Pr_{adult\ clones}$ difference was close to 0, and the difference between $Pr_{perinatal\ AI}$-$Pr_{adult\ AI}$ and $Pr_{perinatal\ clones}$-$Pr_{adult\ clones}$ was highly significant. The plotting of each cloning-related DMR according to the percentage of enriched probes in AI controls (y-axis) and clones (x-axis) revealed a biased distribution toward hypermethylation in AI controls, but only in perinatal animals

(Supplementary Fig. S12F; 70 out of 83 DMRs were hypermethylated in perinatal AI). Similar to age-related DMRs, the difference between $Pr_{perinatal\ AI}$-$Pr_{perinatal\ clones}$ and $Pr_{adult\ AI}$-$Pr_{adult\ clones}$ was highly significant (Supplementary Fig. S12H).

Taken together, these results suggest that nuclear reprogramming interfered with the establishment of specific epigenetic signatures of age, leading to uncoupling of DNA methylation and age. Nuclear reprogramming particularly affected the epigenome of pathological perinatal clones, but had limited effects on the epigenome of clinically normal adult clones.

Pyrosequencing validation

We extended the cohort to a larger set of animals for validation (n=35; Table 1), so as to rule out the possibility that our results were due to the particular behaviour of the four animals in perinatal AI group. Three postnatal AI controls were therefore added, allowing most perinatal clones to be flanked by both younger and older controls, as well as four perinatal clones and two adult clones. Twelve regions were selected for validation, based on their contribution to normal or altered hepatic function and on their wide variety of enrichment patterns, CpG contents, size and position relative to the gene start. Some of them were representative of the global behaviour of age-related DMRs and cloning-related DMRs (1, 3, 4 in Supplementary Fig. S12E and 1 in Supplementary Fig. S12F), whereas some displayed a different behaviour (2 in Supplementary Fig. S12E and 2, 3 in Supplementary Fig. S12F). Overall, the 12 regions represented 115 analysed CpGs (Supplementary Fig. S13A). We used the pyrosequencing of bisulphite-converted DNA to quantify the absolute methylation percentage of individual CpGs[8], and then calculated the average methylation percentage in the considered region of each animal. Nine of the 12 DMRs were validated, in that they displayed a significant positive correlation between pyrosequencing and microarray data. Three DMRs were not validated (DMRs 3, 8 and 12), which is similar to the rates reported by others for MeDIP data[9]. When all the regions validated were considered together, a significant positive correlation was observed between the average percentage of enriched probes at the nine DMRs and the average level of

methylation quantified by pyrosequencing the corresponding 98 CpGs (Supplementary Fig. S13B). The adult AI were hypomethylated in the two sets of data for this particular combination of DMRs. The percentage of enriched probes ranged from 0 to 50% (x-axis) for an absolute methylation level ranging from 45% to 55% (y-axis), suggesting that our MeDIP-chip experiment and analytical pipeline were able to detect small variations between groups in methylated regions, but might be not sensitive enough to highlight regions with low methylation levels (<40%). Interestingly, for all the regions analysed (including those not validated), we observed significantly different pyrosequencing results between groups in at least one of the two cohorts (26 or 35 individuals; Supplementary Fig. S13A). The different specificity for methylation and hydroxymethylation of the two techniques used here may partly account for some of the discrepancies between the microarray data and the pyrosequencing results[10].

Supplementary Fig. S13C-H shows the detailed results relative to six individual DMRs: three age-related DMRs (DMR1-3), two cloning-related DMRs (DMR4-5) and one region belonging to the intersection between age-related DMRs and cloning-related DMRs (DMR6). Overall, the results obtained by MeDIP-chip and pyrosequencing were in good agreement, which validated the microarray data and led to new candidate genes with methylation patterns affected by age and/or cloning in the cattle liver.

Supplementary Discussion

The hypomethylation we observed in our cattle clones deceased in the perinatal period is reminiscent of the hypomethylation reported in abnormal cloned piglet[11]. Whether this hypomethylation arose from incomplete reprogramming of the donor genome, or appeared secondary to the onset of disease, still needs to be determined.

Consistent with the report that normal progression to adulthood and ageing in humans is paralleled by a global loss of methylation[12], we showed that adult AI controls and adult clones were hypomethylated compared to perinatal AI controls. Strikingly, perinatal clones were demethylated at a level similar to that of adults at DMRs, demonstrating that they were epigenetically older than expected for their chronological age. It is proposed that the poor outcome of cloning is related to a persistence of the epigenetic memory of donor cells after nuclear reprogramming[13,14]. Accordingly, it is tempting to speculate that the uncoupling of DNA methylation from age in pathological perinatal clones, which may lead to metabolic conditions unable to meet the requirements of the growing foetus, arose from incomplete reprogramming of the adult donor epigenome.

In contrast to this view, rather than being a manifestation of incomplete donor cell reprogramming, uncoupling of DNA methylation from age could be a consequence of the metabolic disorders appearing in pathological perinatal clones. Several studies, reviewed in[15], reported the demethylation of specific CpGs in liver pathologies such as hepatocellular carcinoma and non-alcoholic fatty liver disease without the intervention of nuclear reprogramming. Furthermore, premature epigenetic ageing has been observed in the livers of obese people[16] and in the leukocytes of patients with type 2 diabetes[17], thus reinforcing the relationship between epigenetic ageing and metabolic disorders.

Supplementary Methods

Methylated DNA immunoprecipitation and microarray hybridization

A Roche-NimbleGen 3x720K microarray targeting the upstream region of most bovine coding and non-coding genes was designed according to the UMD3.1 assembly. An annotation file was downloaded from the Johns Hopkins University Center for Computational Biology FTP site (ftp://ftp.ccb.jhu.edu/pub/data/assembly/Bos_taurus/Bos_taurus_UMD_3.0/annotation/; accessed Aug. 2010). Pseudogenes were filtered out from the file, resulting in a total of 21,296 genes, which were tiled from -2000 to +1360 bp relative to the gene start ("promoters"). 10,866 promoters contained a CpG island. In addition, 22,387 probes covering potentially imprinted loci and surrounding regions (including 209 CpG islands), as well as one region devoid of CpGs, were added to the microarray ("large regions", Supplementary Table S1). To enable the optimal coverage of tiled regions, non-unique probes matching up to 10 times with the genome were included in the design, representing 6.9% of all probes.

DNA extraction from the liver samples, MeDIP and quality controls by PCR were performed as described elsewhere ([18] and Supplementary Fig. S5). In order to prevent any technical biases, the products of 9-10 independent MeDIP experiments were pooled for each animal. 100 ng of this material, as well as 100 ng input DNA, were then subjected to moderate genome amplification (10 cycles; GenomePlex complete WGA kit, Sigma). After purification with the QIAquick PCR purification kit (Qiagen), 1 µg amplified samples were labelled with Cy3 and Cy5 in two separate reactions using a dual-colour DNA labelling kit (Roche-NimbleGen). For each sample, 15 µg labelled MeDIP and 15 µg labelled input were then assembled in both combinations (Cy3-input with Cy5-MeDIP or Cy5-input with Cy3-MeDIP), dried and resuspended in hybridization reagents (hybridization kit, Roche-NimbleGen). Microarrays were hybridized at 42°C for 17-18h in a NimbleGen hybridization system (transcriptomic platform POPS, IPS2, INRA, France) and washed using a wash buffer kit (Roche-NimbleGen) according to the manufacturer's instructions. The hybridization lay-out incorporated

technical dye-swaps for every sample (Supplementary Fig. S5). Fluorescent signals were scanned at 532 nm and 635 nm using an Innoscan 900 scanner (Innopsys) at a 3 µm resolution. Raw data files containing the Cy3 and Cy5 signal quantifications for each probe were generated using Nimblescan 2.6 software (Roche-NimbleGen).

Computation of probe status

Probes with signal enrichment in the MeDIP sample ("enriched probes") were identified using the ChIPmix R package[2,19] as follows. Hybridization data were normalized using an ANOVA model[20]. Data were averaged on the dye-swap to remove dye biases. For each probe, the local CpG percentage was then computed within a 400 bp genomic window centred on the probe position. Finally, ChIPmix was run on the normalized data, with three classes of probes analysed independently: low CpG class (CpGs<1%), intermediate CpG class (1%≤CpGs<4%) and high CpG class (CpGs≥4%). For each group of animals, the percentage of enriched probes at each probe position was visualized using IGV[21].

Chromosome enrichment analysis (Supplementary Fig. S8)

A normalized enrichment per chromosome NEci was calculated for each chromosome c and each individual i as follows:

$\mathrm{NEci} = \frac{\mathrm{Eci}}{\mathrm{Tc}} / \frac{\mathrm{Gi}}{\mathrm{T}}$, where Eci is the number of enriched probes at promoters for chromosome c and individual i; Gi is the total number of enriched probes at promoters for individual i; Tc is the total number of probes at promoters for chromosome c, and T is the total number of probes at promoters. For the local methylation analysis of chromosome 18, the proportion of enriched probes within a sliding window of ten promoters was computed considering all individuals together. This proportion was then compared with the proportion of enriched probes for the whole of chromosome 18 using a Poisson one-sided test. The p-values obtained for all sliding windows were corrected for multiple testing using the Benjamini-Hochberg procedure[22] with the multtest R package. Sliding windows displaying a significant increase/decrease in the proportion of enriched probes were then coloured in

red and blue, respectively (adjusted p-values<0.01). In other cases, sliding windows were displayed in black.

Principal component analysis on methylated DNA immunoprecipitation data (Supplementary Fig. S10A)

A normalized enrichment factor NEpi was calculated for each promoter p and each individual i as follows:

$\mathrm{NEpi} = \mathrm{Epi}\frac{\mathrm{G}}{\mathrm{Gi}}$, where:

Epi =Number of enriched probes for promoter p and individual i;

$\mathrm{Gi} = \sum_{\mathrm{p}=1}^{21296} \mathrm{Epi}$ = Total number of enriched probes at promoters for individual i;

$\mathrm{G} = \frac{1}{26}\sum_{\mathrm{i}=1}^{26}\sum_{\mathrm{p}=1}^{21296} \mathrm{Epi}$ =Mean Gi for all individuals.

PCA was then computed on the NEpi matrix using FactoMineR R package.

Identification of regions of interest

A first set of regions of interest was defined considering two groups, clones and AI controls, in which the 26 animals were classified. Each promoter or large region was screened for an anchor probe displaying enrichment in more than 40% of animals belonging to the same group. From this anchor, the region was then extended to upstream and downstream probes until a probe displaying less than 10% enrichment in any group was encountered. Two consecutive regions of interest separated by fewer than three probes were coalesced into one larger region of interest. After coalescence, regions containing only the anchor probe were eliminated. Indeed, the signal from a single probe could result from cross-hybridization with another genomic region, whereas this probability could be reduced if several probes were locally enriched. A second set of regions of interest was then defined relative to age, according to the same procedure. The two groups considered for this second set were perinatal animals and adults. Because the partitioning of

individuals into the two groups was slightly different for age and cloning, the two sets of regions were not completely equivalent. Consensus regions of interest were obtained by combining them according to the following rules: for regions included in others, the largest one was retained, whereas overlapping regions were concatenated, resulting in a total of 15,885 regions. Regions of interest were then annotated relative to the most upstream start codon of each coding gene and to exon definitions according to the UMD.1 annotation file (ftp://ftp.ccb.jhu.edu/pub/data/assembly/Bos_taurus/Bos_taurus_UMD_3.0/annotation/). All transcripts were considered. Gene ontology (GO) analysis in Supplementary Fig. S9 was performed using the PANTHER database[23].

Identification of differentially methylated regions

DMRs were identified using an R package designed to analyse point patterns [6] which was therefore appropriate for binary data (in the present case: 0=probe not enriched *vs* 1= probe enriched). Three models were built and estimated using the ppm function of the spatstat R package (ANOVA for fitted point process models): one full model took account of both age and cloning (i) and two alternative models took account of age only (ii) or cloning only (iii). Due to the lack of statistical power, the interaction between age and cloning was not considered. To identify cloning-related DMRs and age-related DMRs, the full model was compared with alternative models (ii) and (iii), respectively. All 15,885 regions of interest were tested and the resulting p-values were corrected for multiple testing using the Benjamini-Hochberg procedure[22] with the multtest R package. A given region was considered as a cloning-related DMR if the full model fitted the observations significantly better than alternative model (ii) (adjusted p-value<0.05). Similarly, a given region was considered as an age-related DMR if the full model fitted the observations significantly better than alternative model (iii).

Hierarchical clustering (Supplementary Fig. S12A-B)

Hierarchical clustering was performed separately for age-related DMRs and cloning-related DMRs on the number of enriched probes per DMR. Pearson correlation coefficients were used to compute the distance between samples and ward method was used as linkage function.

Computation of enrichment scores Pi and Pr

For each individual i, the average percentage of enriched probes Pi at all DMRs was calculated separately for age-related DMRs (Pi_{age}) and cloning-related DMRs ($Pi_{cloning}$) as follows:

$\mathrm{Pi} = \frac{1}{\mathrm{n}}\sum_{\mathrm{r}=1}^{\mathrm{n}} \frac{\mathrm{Eri}}{\mathrm{Tr}} \times 100$, where Eri is the number of enriched probes for DMR r and individual i; Tr is the number of probes included in DMR r; and n is the total number of DMRs (n=246 for age-related DMRs and n=83 for cloning-related DMRs). For Supplementary Fig. S11, the correlation between all Pi_{age} and $Pi_{cloning}$ was estimated using Spearman's rank correlation test. For Supplementary Fig. S12C-D, groups were compared using a permutation test for k independent samples (Monte-Carlo sampling of 100,000 permutations, coin R package) followed by pairwise comparisons (1000 permutations; Benjamini-Hochberg correction).

For Supplementary Fig. S12E-H, the mean percentage of enriched probe Pr in each group of animals was calculated as follows for each DMR r:

$\mathrm{Pr} = \frac{1}{\mathrm{m}}\sum_{\mathrm{i}=1}^{\mathrm{m}} \frac{\mathrm{Eri}}{\mathrm{Tr}} \times 100$, where m is the total number of individuals in the group considered (m=4 for perinatal AI controls; m=7 for perinatal clones; m=8 for adult AI controls and m=7 for adult clones). Four Pr were then obtained per DMR: $Pr_{perinatal\ AI}$, $Pr_{perinatal\ clones}$, $Pr_{adult\ AI}$, $Pr_{adult\ clones}$. For age-related DMRs, the $Pr_{perinatal\ AI}$ - $Pr_{adult\ AI}$ differences were compared to the $Pr_{perinatal\ clones}$ - $Pr_{adult\ clones}$ differences using a Wilcoxon test for paired samples. The same test was applied to compare the $Pr_{perinatal\ AI}$ - $Pr_{perinatal\ clones}$ differences with the $Pr_{adult\ AI}$ - $Pr_{adult\ clones}$ differences for cloning-related DMRs.

Pyrosequencing (Supplementary Fig. S13)

Bisulphite conversion was performed on 1 µg liver genomic DNA as described elsewhere[8]. After ethanol precipitation, the DNA pellet was resuspended in 20 µl H_2O. The targeted regions were amplified by PCR with Platinum Taq DNA polymerase (Invitrogen) from 1 µl treated DNA in 50 µl reaction volume, according to the manufacturer's instructions with variable $MgCl_2$ concentrations. The PCR programme was: 3 min. at 94°C followed by 50 cycles of 30 s at 94°C, 1 min. at variable hybridization temperatures, 1 min. at 72°C, with a final extension of 10 min. at 72°C. The primers used to amplify each region are listed in Supplementary Table S2, together with the hybridization temperatures and $MgCl_2$ concentrations. Primers were designed using the MethPrimer program[24]. The reverse primers were biotinylated for all regions except *SLC38A4*, for which the forward primer was biotinylated. 20 µl of the PCR reaction was used as a template for pyrosequencing with 0.3 µM pyrosequencing primer, using the Pyromark Q24 device and Pyromark Gold Q96 reagents (Qiagen). Pyrosequencing primers are listed in Supplementary Table S3. Each CpG was assayed in duplicate, and inconsistent duplicates (more than 5% difference) were repeated. The methylation percentage per CpG was then obtained by calculating the mean of all replicates that passed the quality control of the Pyromark Q24 software.

The average methylation percentage of all CpGs analysed in a given region was computed for each individual. Groups were then compared using a permutation test for k independent samples (Monte-Carlo sampling of 100,000 permutations, coin R package) followed by pairwise comparisons (1000 permutations; Benjamini-Hochberg correction). Correlations related to pyrosequencing data were estimated using Spearman's rank correlation test.

Multivariate analyses: principles and application to our datasets

Three types of multivariate analyses were conducted in this study: PCA (Figure 2B), multiple correspondence analysis (MCA, Figure 1B) and multiple factor analysis (MFA, Figure 4A, 5A).

The phenotypic (pathology, histomorphometric measurements, fatty acid composition) and epigenetic (DMRs) data generated for the purpose of this study constitute complex matrixes combining many variables. The aim of these multivariate analyses is to reduce the n-dimension space formed by the n initial variables to a 2-dimension space in order to allow visualization, while retaining a maximum of information. Each dimension is therefore a combination of the n initial variables that maximizes the variability between individuals. Dimension 1 is set to explain the main part of variance, dimension 2 explains the main part of the remaining variance, etc. In these combinations, a loading is assigned to each variable for each dimension. The more important this loading is for a given dimension, the more the variable contributes to the dispersion of individuals along the dimension.

PCA, MCA and MFA are without *a priori* analyses, meaning that they aim to describe at best the data and not to identify variables that show a contrasted behaviour in the different groups. However, since dimensions 1 and 2 explain the main part of total variance, a discrimination of individuals according to the groups by these dimensions necessarily leads to the conclusion that the groups are indeed the principal source of variability between individuals. In this case, the variables that are different between groups can easily be identified, because they match with those displaying the most important loadings along dimensions 1 and 2.

In PCA, dimensions represent linear combinations of quantitative variables that maximize the variance. PCA was applied to two sets of quantitative variables: normalized enrichment factors NEpi (Supplementary Fig. S10) and percentages of fatty acids (FAs, Figure 2B).

The goals and principles of MCA are very similar to PCA, except that MCA also fits for qualitative variables such as those produced by histopathological analyses[25]. In this study MCA was generated from 7 variables, corresponding to 7 histopathological features with scored categories as modalities (Figure 1B). Dimensions represent combinations of variables that maximize the difference with a theoretical situation where all variables are independent and the categories equally distributed in the different groups. The processing of data also allows a combined representation of both individuals and categories in the same plan. The relative distance of individuals with a category

then indicates that this category is more represented among these individuals than in the remaining population.

The goal of MFA is to integrate several sets of variables measured on the same individuals. In this study, MFA was computed on methylation at age-related DMRs and cloning-related DMRs (DMR set, 282 variables), histomorphometric parameters measured on hepatocytes (Morpho set) and FA composition (FA set). The basis is a particular PCA in which the sets of variables are weighted in order to take them equally into account. MFA therefore generates dimensions that combine variables from the different sets. Originally used to explore sensory and ecology data [26], MFA has recently been applied to omics datasets [27]. The data are processed to allow a representation of all sets of variables in the same plan (as in Figures 4A and 5A). Two individuals, two variables (issued from the same or different sets) or two sets that are strongly correlated appear close together on this plan. The sets and illustrative variable (here: the one-variable set Group summarizing the origin of individuals) are projected on the different dimensions. The coordinates of these projections indicate which dimension provides a partition according to the illustrative variable and which set(s) contribute(s) to this partition the most. Variables of the different sets explaining this partition can be identified based on their correlation with the appropriate dimension.

Supplementary References

1 Weber, M. *et al.* Chromosome-wide and promoter-specific analyses identify sites of differential DNA methylation in normal and transformed human cells. *Nature genetics* **37**, 853-862, doi:10.1038/ng1598 (2005).

2 Martin-Magniette, M. L., Mary-Huard, T., Berard, C. & Robin, S. ChIPmix: mixture model of regressions for two-color ChIP-chip analysis. *Bioinformatics* **24**, i181-186, doi:10.1093/bioinformatics/btn280 (2008).

3 Weber, M. *et al.* Distribution, silencing potential and evolutionary impact of promoter DNA methylation in the human genome. *Nature genetics* **39**, 457-466, doi:10.1038/ng1990 (2007).

4 Chaligne, R. & Heard, E. X-chromosome inactivation in development and cancer. *FEBS letters* **588**, 2514-2522, doi:10.1016/j.febslet.2014.06.023 (2014).

5 Rauch, T. A., Wu, X., Zhong, X., Riggs, A. D. & Pfeifer, G. P. A human B cell methylome at 100-base pair resolution. *Proceedings of the National Academy of Sciences of the United States of America* **106**, 671-678, doi:10.1073/pnas.0812399106 (2009).

6 Baddeley, A. & Turner, R. spatstat: An R Package for Analyzing Spatial Point Patterns. *Journal of Statistical Software* **12** (2005).

7 Schubeler, D. Function and information content of DNA methylation. *Nature* **517**, 321-326, doi:10.1038/nature14192 (2015).

8 Dupont, J. M., Tost, J., Jammes, H. & Gut, I. G. De novo quantitative bisulfite sequencing using the pyrosequencing technology. *Analytical biochemistry* **333**, 119-127, doi:10.1016/j.ab.2004.05.007 (2004).

9 Radford, E. J. *et al.* In utero effects. In utero undernourishment perturbs the adult sperm methylome and intergenerational metabolism. *Science* **345**, 1255903, doi:10.1126/science.1255903 (2014).

10 Jin, S. G., Kadam, S. & Pfeifer, G. P. Examination of the specificity of DNA methylation profiling techniques towards 5-methylcytosine and 5-hydroxymethylcytosine. *Nucleic acids research* **38**, e125, doi:10.1093/nar/gkq223 (2010).

11 Li, G. *et al.* Dysregulation of genome-wide gene expression and DNA methylation in abnormal cloned piglets. *BMC genomics* **15**, 811, doi:10.1186/1471-2164-15-811 (2014).

12 Heyn, H. *et al.* Distinct DNA methylomes of newborns and centenarians. *Proceedings of the National Academy of Sciences of the United States of America* **109**, 10522-10527, doi:10.1073/pnas.1120658109 (2012).

13 Firas, J., Liu, X. & Polo, J. M. Epigenetic memory in somatic cell nuclear transfer and induced pluripotency: evidence and implications. *Differentiation; research in biological diversity* **88**, 29-32, doi:10.1016/j.diff.2014.09.001 (2014).

14 Niemann, H. Epigenetic reprogramming in mammalian species after SCNT-based cloning. *Theriogenology* **86**, 80-90, doi:10.1016/j.theriogenology.2016.04.021 (2016).

15 Mann, D. A. Epigenetics in liver disease. *Hepatology* **60**, 1418-1425, doi:10.1002/hep.27131 (2014).

16 Horvath, S. *et al.* Obesity accelerates epigenetic aging of human liver. *Proceedings of the National Academy of Sciences of the United States of America* **111**, 15538-15543, doi:10.1073/pnas.1412759111 (2014).

17 Toperoff, G. *et al.* Premature aging of leukocyte DNA methylation is associated with type 2 diabetes prevalence. *Clinical epigenetics* **7**, 35, doi:10.1186/s13148-015-0069-1 (2015).

18 Kiefer, H. Genome-Wide Analysis of Methylation in Bovine Clones by Methylated DNA Immunoprecipitation (MeDIP). *Methods in molecular biology* **1222**, 267-280, doi:10.1007/978-1-4939-1594-1_20 (2015).

19 Team, R. C. R: A language and environment for statistical computing. *R Foundation for Statistical Computing* **Vienna**, http://www.R-project.org/ (2014).
20 Turck, F. *et al.* Arabidopsis TFL2/LHP1 specifically associates with genes marked by trimethylation of histone H3 lysine 27. *PLoS genetics* **3**, e86, doi:10.1371/journal.pgen.0030086 (2007).
21 Robinson, J. T. *et al.* Integrative genomics viewer. *Nature biotechnology* **29**, 24-26, doi:10.1038/nbt.1754 (2011).
22 Benjamini, Y. & Hochberg, Y. Controlling the False Discovery Rate: A Practical and Powerful Approach to Multiple Testing. *Journal of the Royal Statistical Society. Series B (Methodological)* **57**, 289-300 (1995).
23 Mi, H., Muruganujan, A. & Thomas, P. D. PANTHER in 2013: modeling the evolution of gene function, and other gene attributes, in the context of phylogenetic trees. *Nucleic acids research* **41**, D377-386, doi:10.1093/nar/gks1118 (2013).
24 Li, L. C. & Dahiya, R. MethPrimer: designing primers for methylation PCRs. *Bioinformatics* **18**, 1427-1431 (2002).
25 Meyer, N., Ferlicot, S., Vieillefond, A., Peyromaure, M. & Vielh, P. [Contribution of multiple correspondence analysis in histopathology]. *Annales de pathologie* **24**, 149-160 (2004).
26 Escofier, B. & Pagès, J. Multiple factor analysis (AFMULT package). *Computational Statistics & Data Analysis* **18**, 121-140 (1994).
27 de Tayrac, M., Le, S., Aubry, M., Mosser, J. & Husson, F. Simultaneous analysis of distinct Omics data sets with integration of biological knowledge: Multiple Factor Analysis approach. *BMC genomics* **10**, 32, doi:10.1186/1471-2164-10-32 (2009).

Supplementary Figures

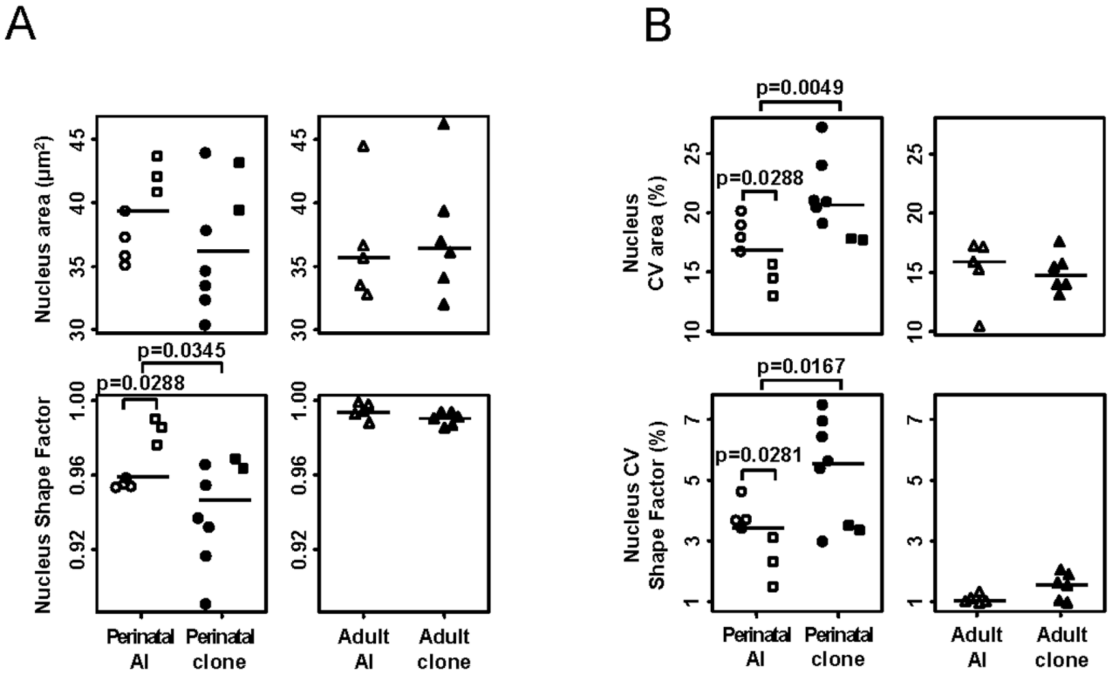

Supplementary Figure S1. Histomorphometric measures are normal in adult clones. For each animal, the area and a shape factor representing circularity were measured on at least 200 nuclei of hepatocytes and the mean (A) and coefficient of variation (CV) (B) were calculated. The CV is indicative of cell heterogeneity in the tissue. Each dot represents one animal. Median values are indicated by horizontal lines. Open dots: AI controls; filled dots: clones; triangles: adults. The scale is identical for perinatal animals and adults.

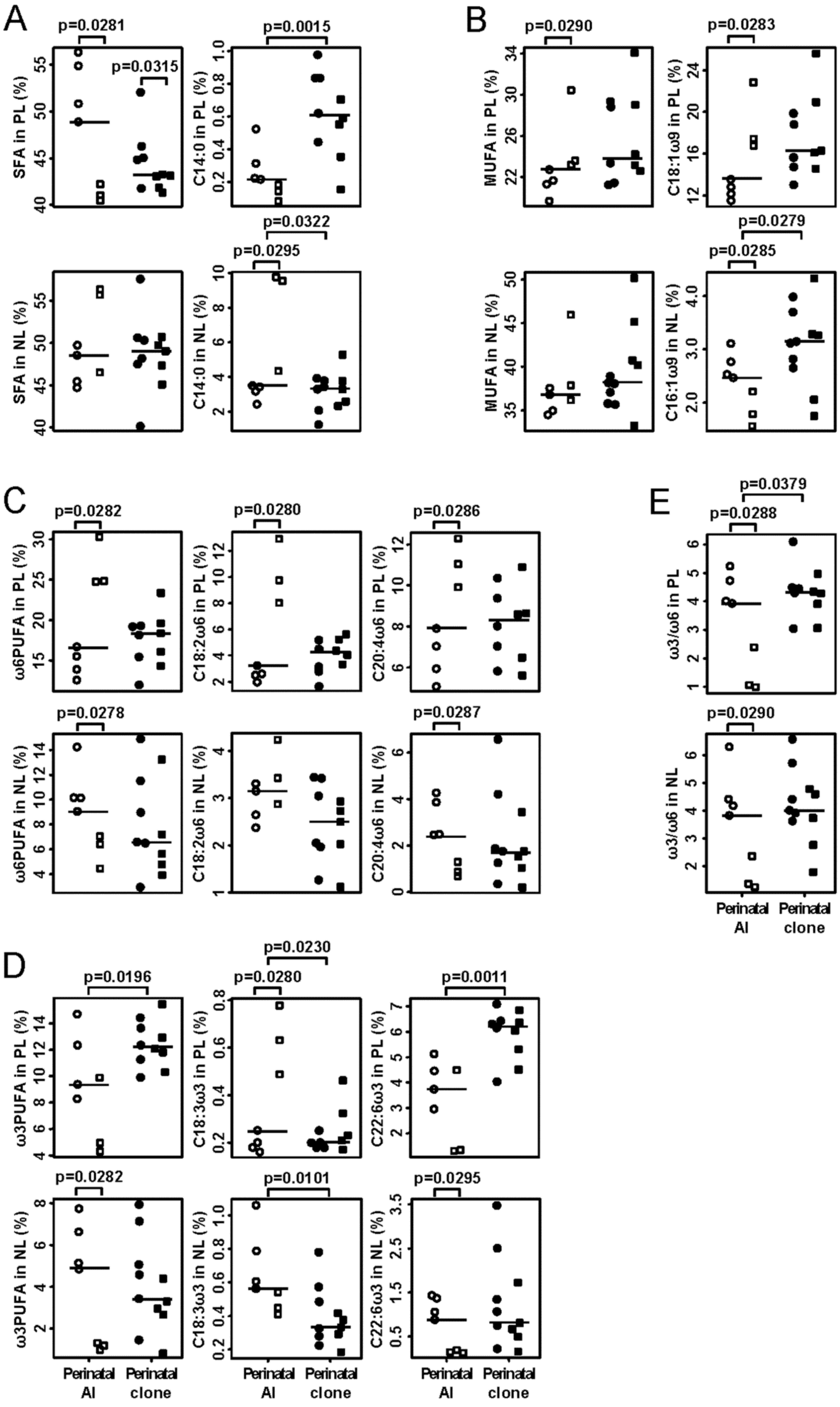
A
p=0.0281
p=0.0315
SFA in PL (%)
p=0.0015
C14:0 in PL (%)
p=0.0322
p=0.0295
SFA in NL (%)
C14:0 in NL (%)
B
p=0.0290
MUFA in PL (%)
p=0.0283
C18:1ω9 in PL (%)
p=0.0279
p=0.0285
MUFA in NL (%)
C16:1ω9 in NL (%)
C
p=0.0282
ω6PUFA in PL (%)
p=0.0280
C18:2ω6 in PL (%)
p=0.0286
C20:4ω6 in PL (%)
p=0.0278
ω6PUFA in NL (%)
C18:2ω6 in NL (%)
p=0.0287
C20:4ω6 in NL (%)
E
p=0.0379
p=0.0288
ω3/ω6 in PL
p=0.0290
ω3/ω6 in NL
Perinatal AI
Perinatal clone
D
p=0.0196
ω3PUFA in PL (%)
p=0.0230
p=0.0280
C18:3ω3 in PL (%)
p=0.0011
C22:6ω3 in PL (%)
p=0.0282
ω3PUFA in NL (%)
p=0.0101
C18:3ω3 in NL (%)
p=0.0295
C22:6ω3 in NL (%)
Perinatal AI
Perinatal clone

Supplementary Figure S2. Fatty acid (FA) composition of the liver in perinatal animals (previous page). The FA composition is expressed as the percentage of total FAs in the phospholipids or neutral lipid. Each dot represents one animal. Open circles: AI foetuses; open squares: AI calves; black circles: cloned foetuses; black squares: cloned calves. Median values are indicated by horizontal lines. Significant differences between AI and clones and between prenatal and postnatal animals are indicated ($p<0.05$, permutation test). (A) Total saturated FAs (SFAs) and one individual SFA (myristic acid C14:0). (B) Total monounsaturated FAs (MUFAs) and one individual MUFA (C18.1ω9). (C) Total omega 6 polyunsaturated FAs (ω6PUFAs) and two individual ω6PUFAs (precursor C18:2ω6 and active metabolite C20:4ω6 (arachidonic acid)). (D) Total omega 3 PUFAs (ω3PUFAs) and two individual ω3PUFAs (precursor C18:3ω3 and active metabolite C22:6ω3 (docosahexaenoic acid)). (E) Ratio between ω3PUFAs and ω6PUFAs. PL: phospholipids, NL: neutral lipids.

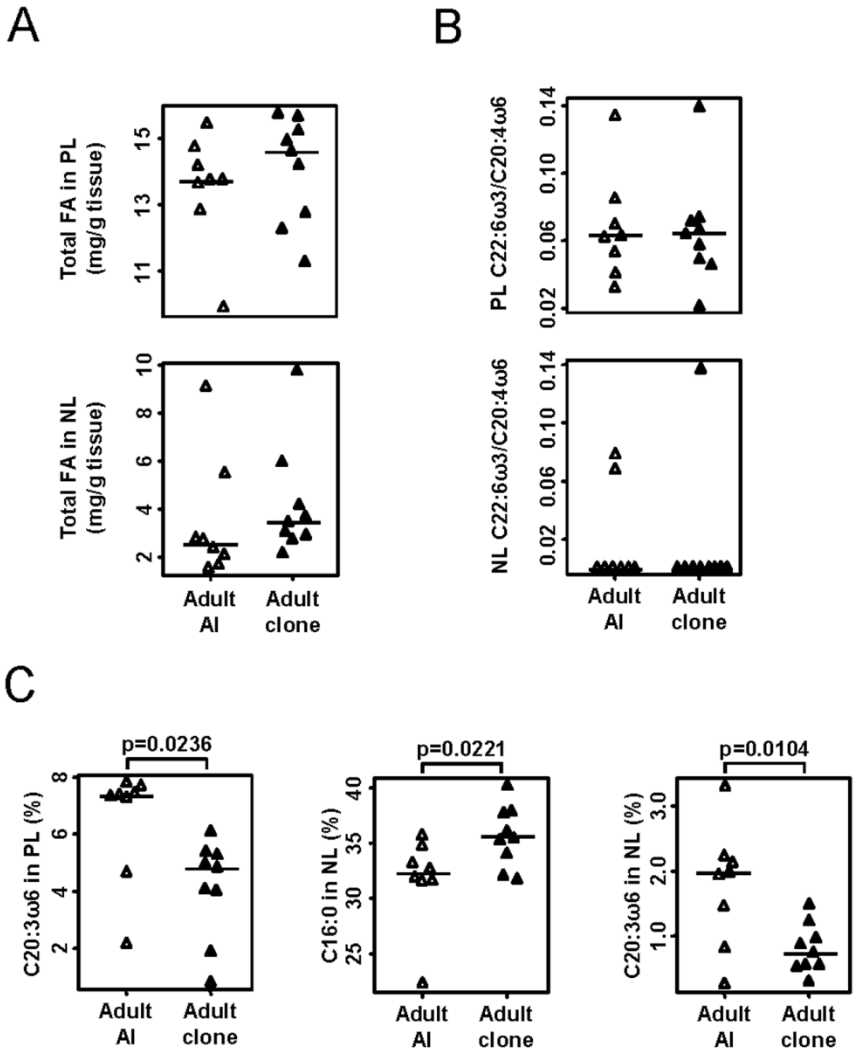

Supplementary Figure S3. FA composition of the liver in adults. Each dot represents one animal. Open triangles: adult AI controls; filled triangles: adult clones. Median values are indicated by horizontal lines. (A) The total amount of FAs was measured by gas chromatography in the phospholipids (PL) and neutral lipids (NL) and expressed as mg/g liver. (B) The ratio between C22:6ω3 and C20:4ω6 was determined in the phospholipids and neutral lipids of each animal. (C) Individual FAs showing significant differences between adult AI and adult clones are represented ($p<0.05$, permutation test).

A

	Perinatal AI	Perinatal clones
Nucleus area (µm²)	0.80	0.02
Nucleus Shape Factor	0.73	0.41
Nucleus CV area (%)	-0.73	-0.74
Nucleus CV Shape Factor (%)	-0.87	-0.25
Cell area (µm²)	-0.11	0.38
Cell Shape Factor	-0.13	-0.19
Cell CV area (%)	-0.40	0.58
Cell CV Shape Factor (%)	0.00	0.09

B

	Phospholipids	
	Perinatal AI	Perinatal clones
C14:0 (%)	-0.80	-0.70
C16:0 (%)	-0.87	-0.87
C18:0 (%)	-0.95	0.39
C20:0 (%)	-0.98	0.47
Total SFA (%)	-0.87	-0.50
C16:1ω7 (%)	-0.49	-0.04
C18:1ω9 (%)	0.65	0.30
Total MUFA (%)	0.58	0.29
C18:3ω3 (%)	0.55	0.29
C20:5ω3 (%)	-0.18	-0.15
C22:5ω3 (%)	-0.58	0.01
C22:6ω3 (%)	-0.05	-0.16
Total ω3PUFA (%)	-0.18	-0.01
C18:2ω6 (%)	0.76	0.25
C18:3ω6 (%)	-0.90	0.39
C20:3ω6 (%)	-0.45	0.21
C20:4ω6 (%)	0.87	-0.27
C22:4ω6 (%)	0.87	0.13
Total ω6PUFA (%)	0.84	0.00
Total FA (mg/g)	0.95	0.04

C

	Neutral lipids	
	Perinatal AI	Perinatal clones
C14:0 (%)	0.65	0.06
C15:0 (%)	-0.95	-0.19
C16:0 (%)	0.65	0.08
C18:0 (%)	-0.98	-0.25
Total SFA (%)	0.33	-0.22
C16:1ω7 (%)	-0.24	0.61
C18:1ω7 (%)	-0.80	0.00
C14:1ω9 (%)	-0.04	0.49
C15:1ω9 (%)	-0.92	0.03
C16:1ω9 (%)	-0.84	-0.26
C18:1ω9 (%)	0.73	0.21
Total MUFA (%)	0.55	0.37
C18:3ω3 (%)	-0.80	-0.58
C20:5ω3 (%)	-0.55	-0.66
C22:5ω3 (%)	-0.55	-0.38
C22:6ω3 (%)	-0.57	-0.39
Total ω3PUFA (%)	-0.58	-0.58
C18:2ω6 (%)	0.44	-0.50
C18:3ω6 (%)	-0.84	0.26
C20:3ω6 (%)	-0.73	-0.21
C20:4ω6 (%)	-0.65	-0.36
C22:4ω6 (%)	-0.09	0.21
Total ω6PUFA (%)	-0.69	-0.38
Total FA (mg/g)	0.56	-0.15

Supplementary Figure S4. Correlations between phenotype and chronological age in the livers of perinatal animals (previous page). Chronological age is expressed as the interval in days between conception (AI for controls, nuclear transfer for clones) and death/necropsy. Spearman's rank correlation coefficients for the correlations between chronological age and quantitative measurements are listed in the tables for perinatal AI and perinatal clones. The coefficients are underlined for significant correlations (Spearman's rank correlation test; $p<0.05$) and shown in italics for non-significant correlations ($p\geq0.05$). (A) For each animal, the area and a shape factor representing circularity were measured on at least 200 nuclei of hepatocytes (Nucleus) and on 35 hepatocytes (Cell). The mean and CV were then calculated. The correlations between these measurements and chronological age are indicated in the table. (B, C) Correlations between chronological age and FA composition in phospholipids (B) and neutral lipids (C). The FAs that were present in very small amount are not represented individually here. PL: phospholipids, NL: neutral lipids; SFA: saturated FAs; MUFA: monounsaturated FAs; ω3PUFA and ω6PUFA: omega 3 and omega 6 polyunsaturated FAs.

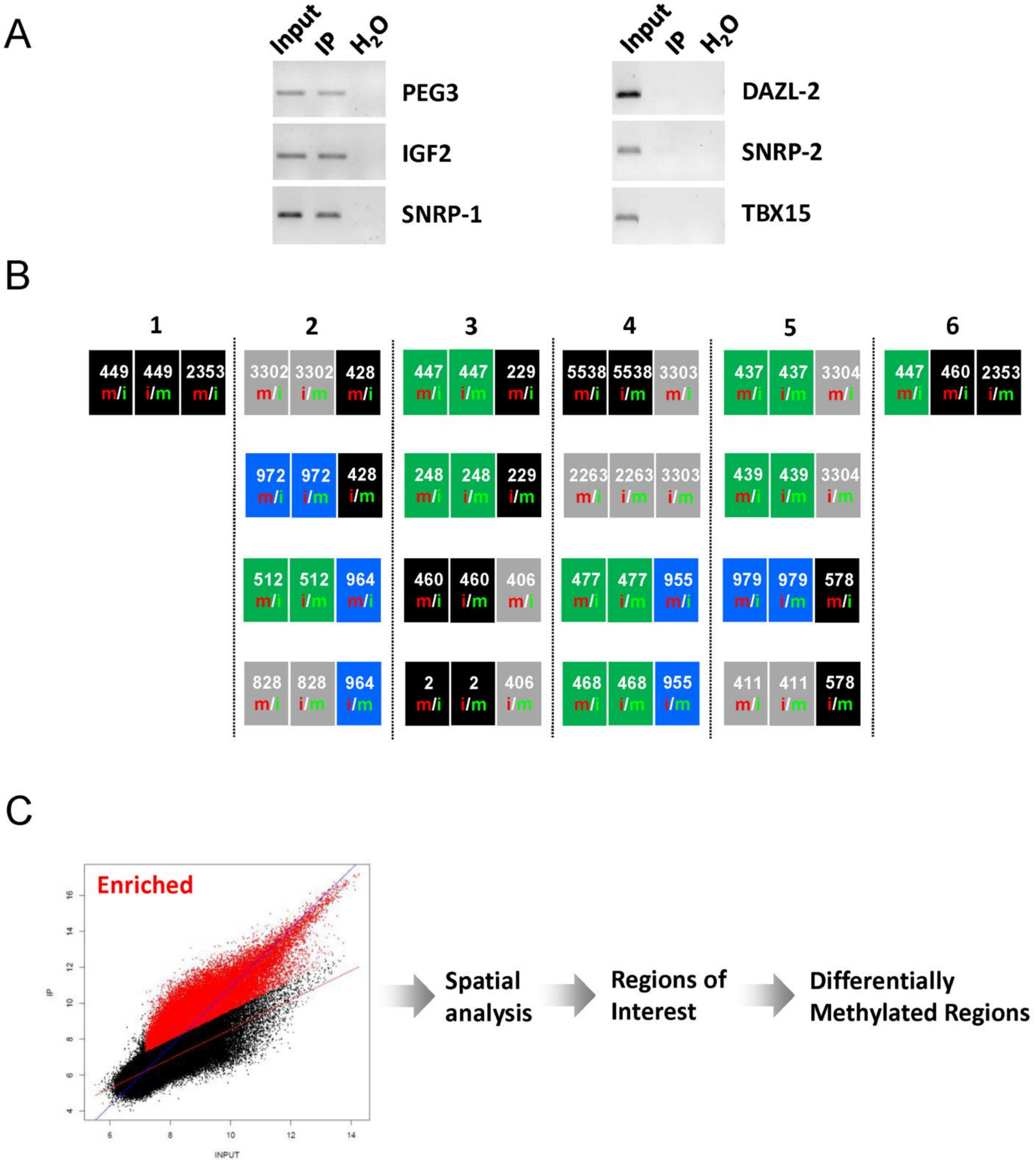
A
Input
IP
H2O
PEG3
IGF2
SNRP-1
DAZL-2
SNRP-2
TBX15
B
1
2
3
4
5
6
449 m/i
449 i/m
2353 m/i
3302 m/i
3302 i/m
428 m/i
972 m/i
972 i/m
428 i/m
512 m/i
512 i/m
964 m/i
828 m/i
828 i/m
964 i/m
447 m/i
447 i/m
229 m/i
248 m/i
248 i/m
229 i/m
460 m/i
460 i/m
406 m/i
2 m/i
2 i/m
406 i/m
5538 m/i
5538 i/m
3303 m/i
2263 m/i
2263 i/m
3303 i/m
477 m/i
477 i/m
955 m/i
468 m/i
468 i/m
955 i/m
437 m/i
437 i/m
3304 m/i
439 m/i
439 i/m
3304 i/m
979 m/i
979 i/m
578 m/i
411 m/i
411 i/m
578 i/m
447 m/i
460 m/i
2353 i/m
C
Enriched
IP
INPUT
Spatial analysis
Regions of Interest
Differentially Methylated Regions

Supplementary Figure S5. MeDIP-chip: experimental design and data analysis (previous page). (A) PCR control of the MeDIP samples using primers specific for methylated regions (left panel) or unmethylated regions/regions devoid of CpGs (right panel). The sequences of the primers and the PCR conditions are listed in[18]. The primer pairs used to amplify *PEG3*, *IGF2*, *SNRP-1*, *DAZL-2*, *SNRP-2* and *TBX15* are PEG3_F1 and PEG3_R1, IGF2_F1 and IGF2_R1, SNRP_F1 and SNRP_R1, DAZL_F2 and DAZL_R2, SNRP_F2 and SNRP_R2, TBX15_F1 and TBX15_R1, respectively. IP: immunoprecipitated DNA. (B) Hybridization layout. Six independent hybridizations, numbered 1 to 6, were performed. The blue, grey, black and green boxes represent microarray chambers hybridized with samples from perinatal AI controls, perinatal clones, adult AI controls, adult clones, respectively. The individuals were randomly assigned to the microarrays and the dye-swap repetitions were hybridized on adjacent chambers whenever possible. Hybridizations were repeated for two chambers showing uneven signal (hybridization #6). m: MeDIP; i: input. The green and red letters indicate Cy3 and Cy5 signal, respectively. (C) Bioinformatics and statistical pipeline. After normalization, probes with signal enrichment in the MeDIP sample were identified using the ChIPmix software for all individuals[2]. Probes with a low, intermediate, and high CpG content were treated separately (see Supplementary Methods and Supplementary Fig. S5). The image shows a representative IP-input plot for one probe class and for one individual. Each dot represents one probe. Probes with signal enrichment in the MeDIP sample (“enriched”) are shown in red. A spatial analysis of the enriched probes was next performed, and regions of interest were delineated. Comparison of the proportions of enriched probes between the four groups then led to the identification of DMRs.

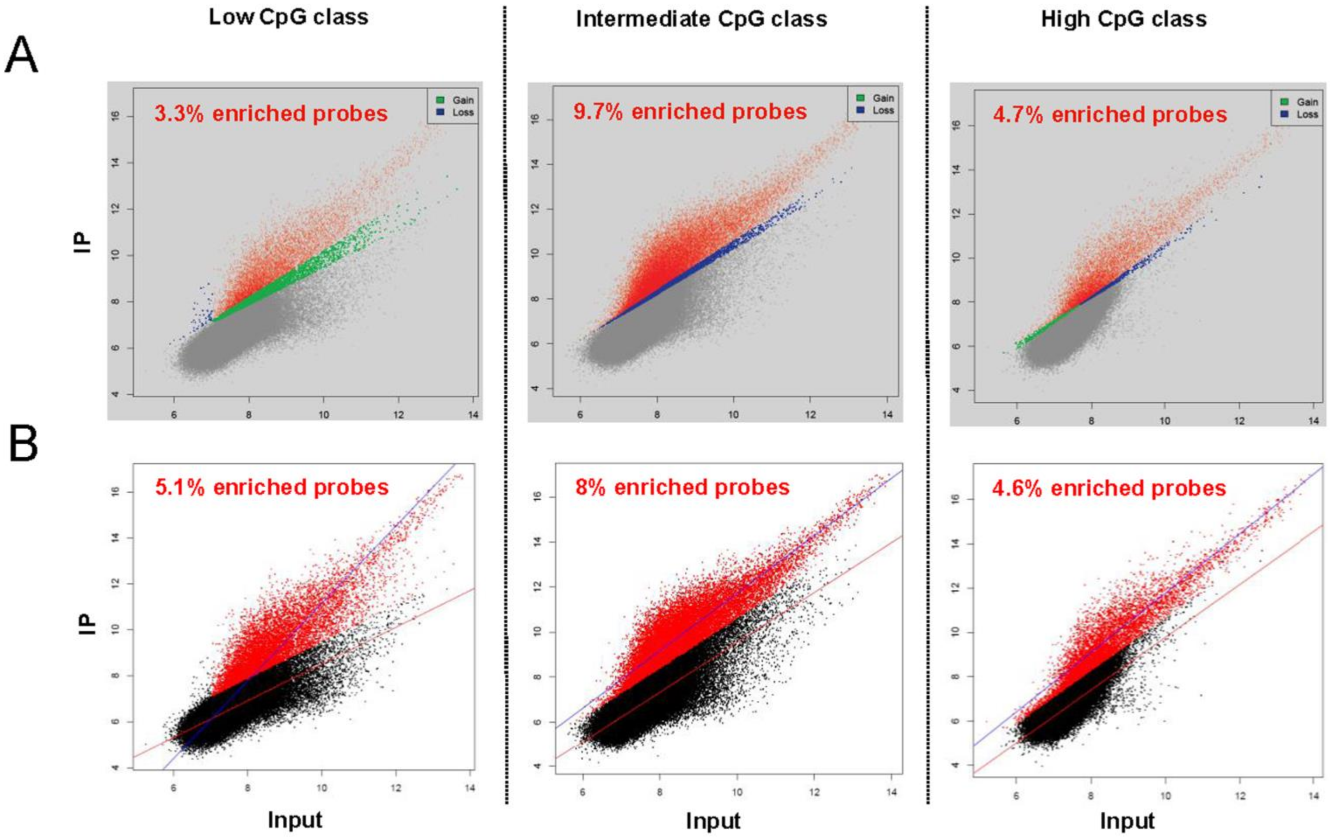

Supplementary Figure S6. Refinement of the ChIPmix analysis by taking account of the probe CpG content. Each image shows an IP-input plot for one representative individual (3302) and for the three probe classes: low, intermediate and high CpG content. Each dot represents one probe. Probes with signal enrichment in the MeDIP sample ("enriched probes") are shown in red. (A) Enriched probes identified in an unique ChIPmix analysis where all the probes were treated together and independent of their CpG content. The percentage of enriched probes in each probe class is indicated in red. The enriched probes that have been gained (green) and lost (blue) in (B) are represented. (B) Final result obtained when the ChIPmix program was run independently for the three probe classes. The percentage of enriched probes has increased in the low CpG class, whereas it has decreased in the intermediate CpG class. It remained unchanged in the high CpG class.

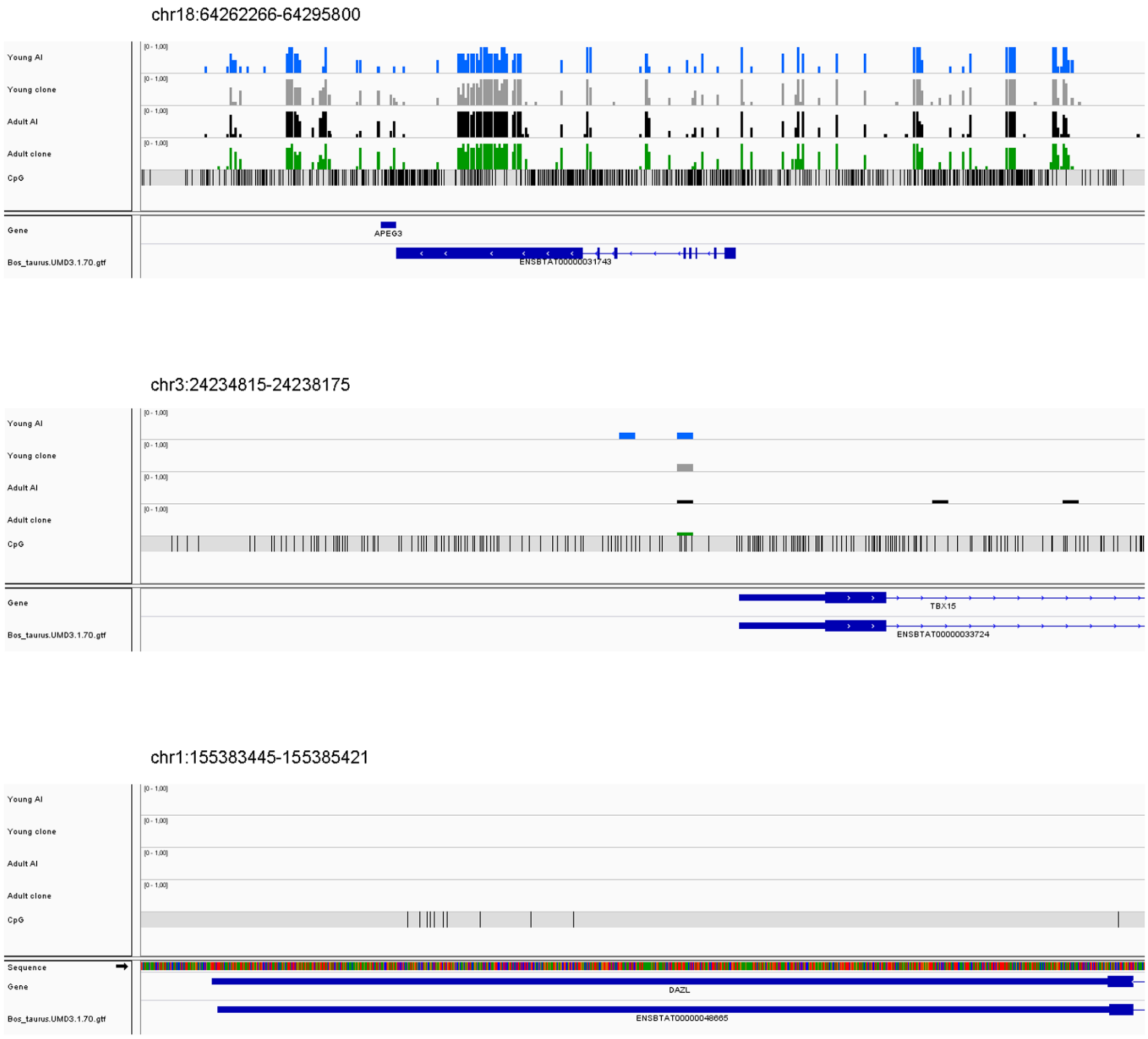

Supplementary Figure S7. Positive and negative controls for MeDIP-chip. IGV browser view of three regions targeted by the microarray. The blue, grey, black and green bar charts represent the proportion of enriched probes at each probe position for the perinatal AI, perinatal clone, adult AI, adult clone groups, respectively. The "CpG" track shows the positions of CpGs. Upper panel, *PEG3* large region (chr18:64262266-64295800); middle panel, *TBX15* promoter (chr3:24234815-24238175) and lower panel, *DAZL* exon 11, CpG poor (chr1:155383445-155385421).

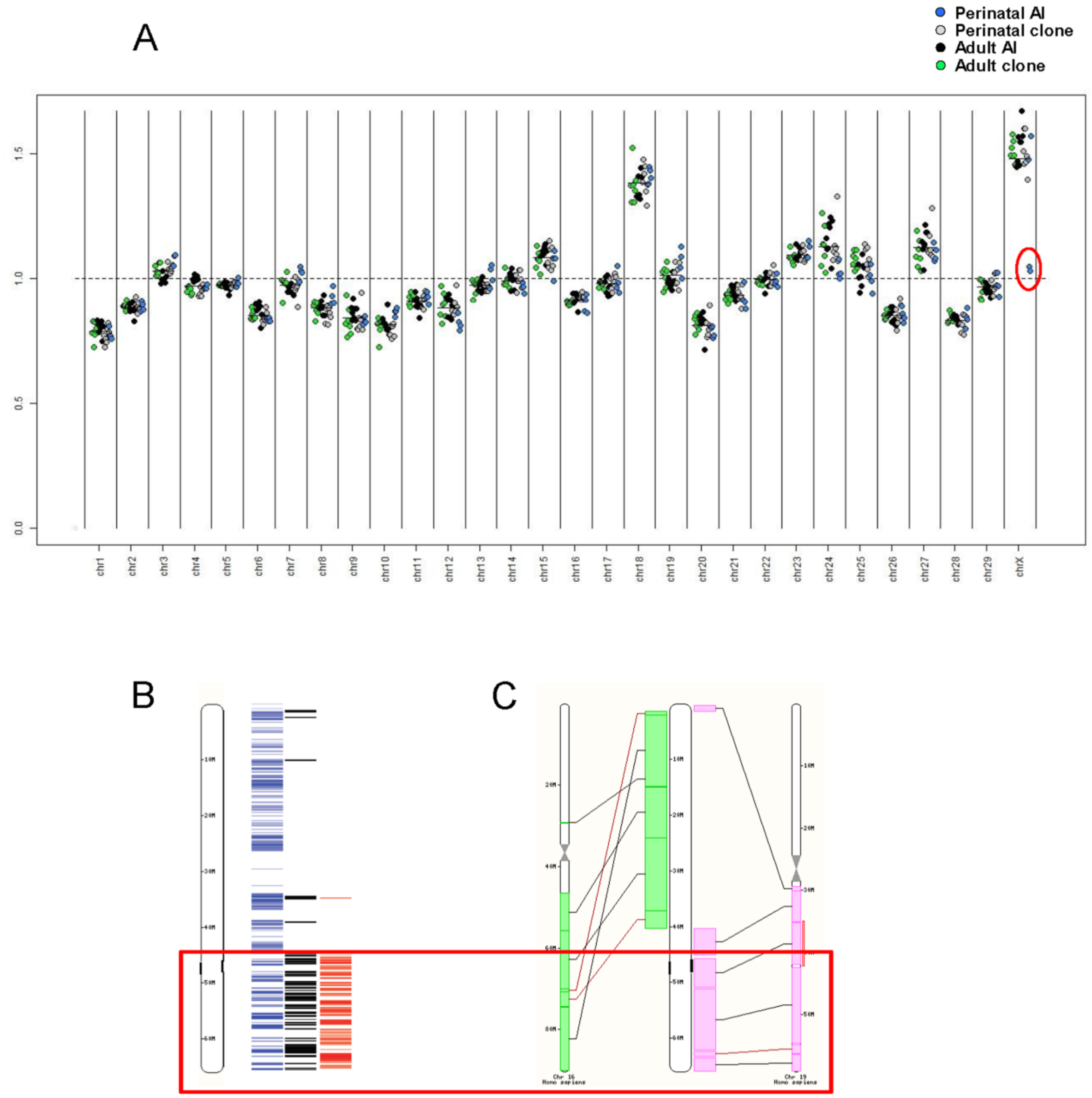

Supplementary Figure S8. Relative methylation enrichment per chromosome. (A) For each individual i and each chromosome c, a normalized enrichment NEci was calculated (see Supplementary Methods). The plot shows this relative enrichment (y-axis) for perinatal AI controls (blue), perinatal clones (grey), adult AI controls (black) and adult clones (green). The two males are circled in red. (B) Local methylation of chromosome 18. The chromosome was divided into sliding windows of ten consecutive promoters. The red bars indicate sliding windows with a significant

higher proportion of enriched probes than the whole of chromosome 18 (local hypermethylation). The blue bars indicate sliding windows with a significant lower proportion of enriched probes than the whole of chromosome 18 (local hypomethylation). In other cases, the sliding window is shown in black. (C) Synteny between Cow chromosome 18 and Human (from: http://www.ensembl.org/Bos_taurus/Location/Synteny?r=18). The red box indicates the emplacement of the hypermethylated region.

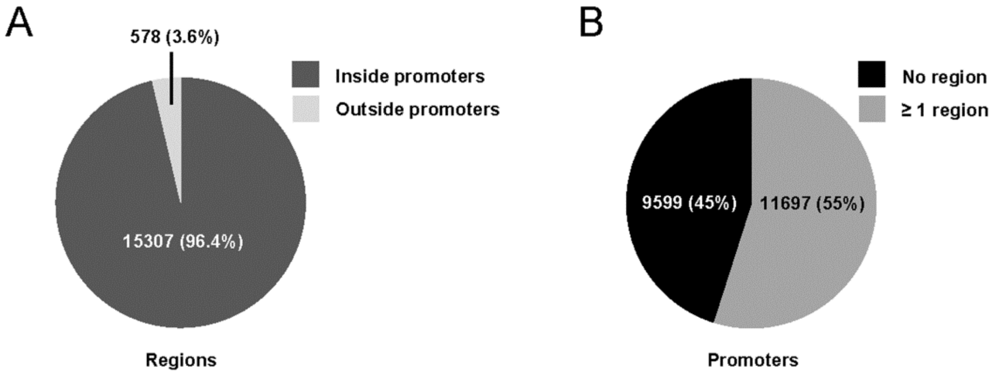

C

Biological process	Gene count	Fold enrichment	p-value
Nitric oxide biosynthetic process	6	2.68	2.67E-02
Unsaturated fatty acid biosynthetic process	7	2.5	2.43E-02
Glycolysis	16	2.2	3.44E-03
Tricarboxylic acid cycle	11	1.97	2.80E-02
Cellular amino acid catabolic process	23	1.52	3.47E-02
Cholesterol metabolic process	35	1.46	2.09E-02
Steroid metabolic process	74	1.45	1.52E-03
Cellular amino acid metabolic process	109	1.4	4.87E-04
Monosaccharide metabolic process	55	1.39	1.07E-02
Negative regulation of apoptotic process	36	1.38	3.62E-02
Generation of precursor metabolites and energy	105	1.35	1.75E-03
Visual perception	78	1.32	9.94E-03
Cellular component morphogenesis	169	1.31	3.95E-04
Respiratory electron transport chain	78	1.27	2.34E-02
Protein glycosylation	62	1.26	4.33E-02
Fatty acid metabolic process	75	1.22	4.78E-02
Carbohydrate metabolic process	191	1.22	3.54E-03
Lipid metabolic process	306	1.22	3.06E-04
Anatomical structure morphogenesis	194	1.2	7.49E-03
Translation	165	1.19	1.51E-02
Cation transport	193	1.19	1.04E-02
Ion transport	237	1.17	7.66E-03
Cellular protein modification process	429	1.16	8.36E-04
Cellular component organization or biogenesis	438	1.15	1.26E-03
Cellular component organization	400	1.15	2.19E-03
Protein metabolic process	883	1.12	1.62E-04
Cellular process	2015	1.06	8.25E-04
Primary metabolic process	2011	1.06	1.34E-03
Metabolic process	2430	1.05	3.67E-04
RNA metabolic process	551	0.93	4.63E-02
Transcription from RNA polymerase II promoter	389	0.92	4.98E-02
Regulation of nucleobase-containing compound metabolic process	367	0.9	1.67E-02
Transcription, DNA-dependent	420	0.89	7.61E-03
Cell adhesion	123	0.85	3.52E-02
Immune response	130	0.84	2.46E-02
Sensory perception of chemical stimulus	59	0.73	6.99E-03
Heart development	36	0.68	9.79E-03
B cell mediated immunity	27	0.58	1.38E-03
Natural killer cell activation	18	0.5	6.64E-04

Supplementary Figure S9. Characterization of the regions of interest. (A) Pie chart showing the origins of the regions of interest. The 578 regions that do not belong to a promoter were located in the 12 large regions. (B) Contribution of promoters to the regions of interest. The 15,307 regions of interest that belong to a promoter were allocated to 11,695 promoters, whereas 9,598 promoters did not contain any region. (C) GO analysis of promoters including at least one region of interest using PANTHER. Terms of biological processes showing statistical over-representation or under-representation are indicated.

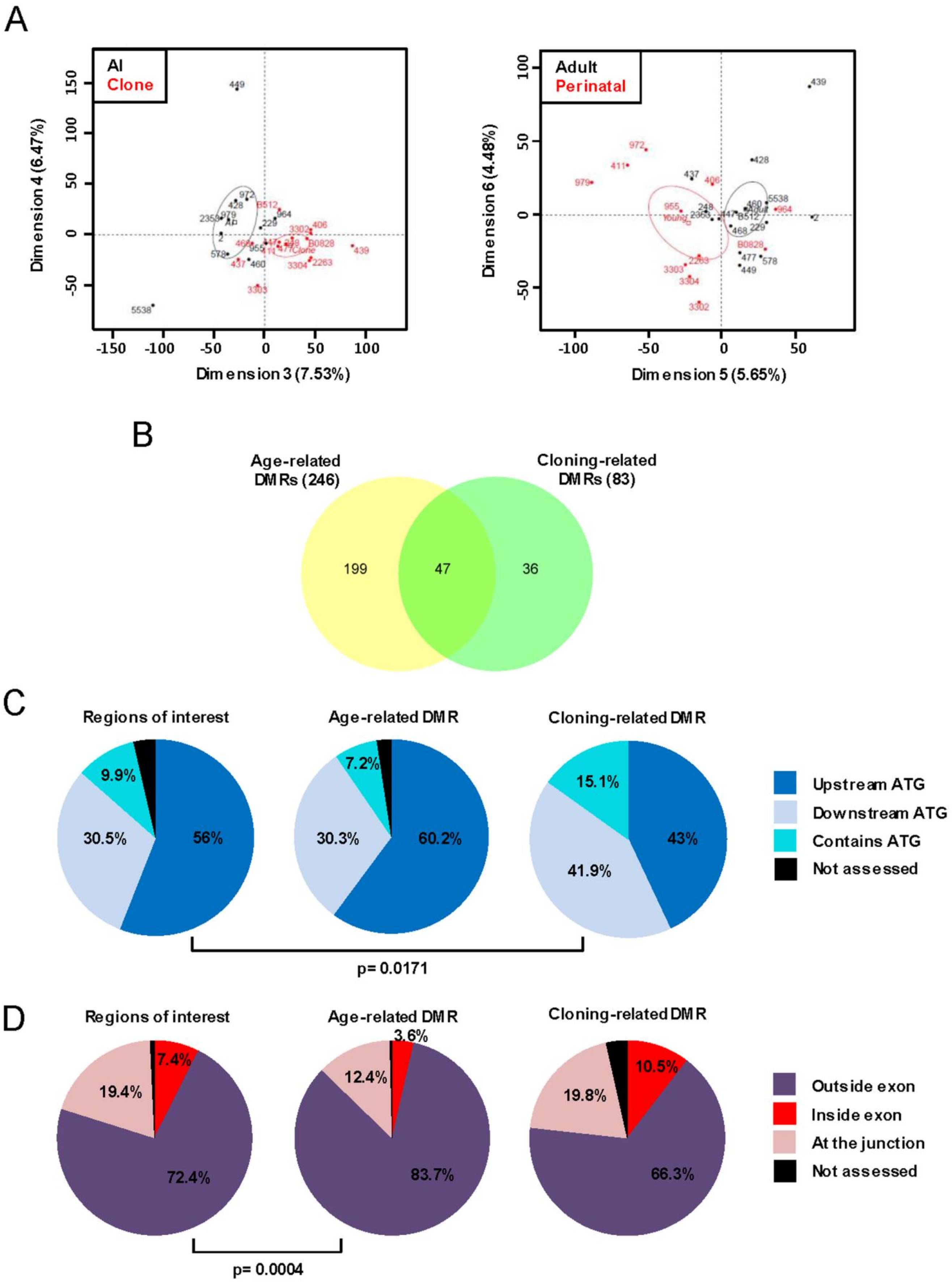
A
AI
Clone
Dimension 4 (6.47%)
Dimension 3 (7.53%)
Adult
Perinatal
Dimension 6 (4.48%)
Dimension 5 (5.65%)
B
Age-related DMRs (246)
Cloning-related DMRs (83)
199
47
36
C
Regions of interest
Age-related DMR
Cloning-related DMR
9.9%
30.5%
56%
7.2%
30.3%
60.2%
15.1%
41.9%
43%
Upstream ATG
Downstream ATG
Contains ATG
Not assessed
p= 0.0171
D
Regions of interest
Age-related DMR
Cloning-related DMR
7.4%
19.4%
72.4%
3.6%
12.4%
83.7%
10.5%
19.8%
66.3%
Outside exon
Inside exon
At the junction
Not assessed
p= 0.0004

Supplementary Figure S10. Intricate relationships between age and cloning in the determination of liver DNA methylation patterns (previous page). (A) For each individual i and each promoter p, a normalized number of enriched probes NEpi was computed (see Supplementary Methods) and PCA was run on the resulting matrix. Left panel: barplot of the contribution of each dimension to total variance; middle panel: discrimination of clones (red) and AI controls (black) by dimension 3; right panel: discrimination of perinatal animals (red) and adults (black) by dimension 5. (B) Venn diagram showing the age-related (yellow) and cloning-related (green) DMRs. (C, D) Regions of interest belonging to promoters were assigned to three categories depending on their position relative to the most upstream ATG (C) and to exons (D). The pie charts show the distributions of regions of interest (left), age-related DMRs (middle) and cloning-related DMRs (right). Significant differences from the distribution of regions of interest are indicated ($p<0.05$, Chi-squared test on the assessed regions). Not assessed: no ATG, and no information on exon/intron localization.

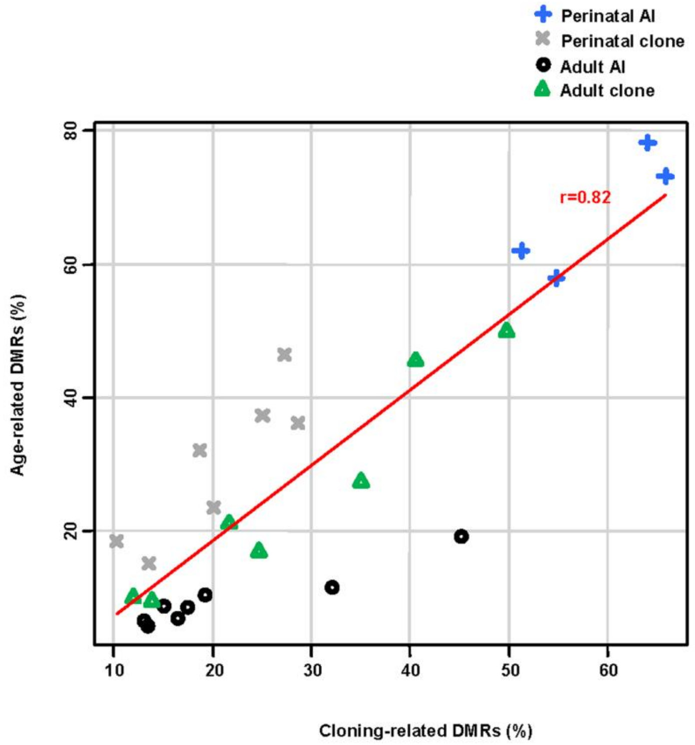

Supplementary Figure S11. Correlation between age-related DMRs and cloning-related DMRs. Each dot represents one animal. The coordinates of each dot is given by the average percentage of enriched probes Pi in the two sets of DMRs for the considered animal (Pi_{age} and $Pi_{cloning}$). The least squares line of best fit and the Spearman's rank correlation coefficient are indicated. The correlation is highly significant (p<5e-5; Spearman's rank correlation test).

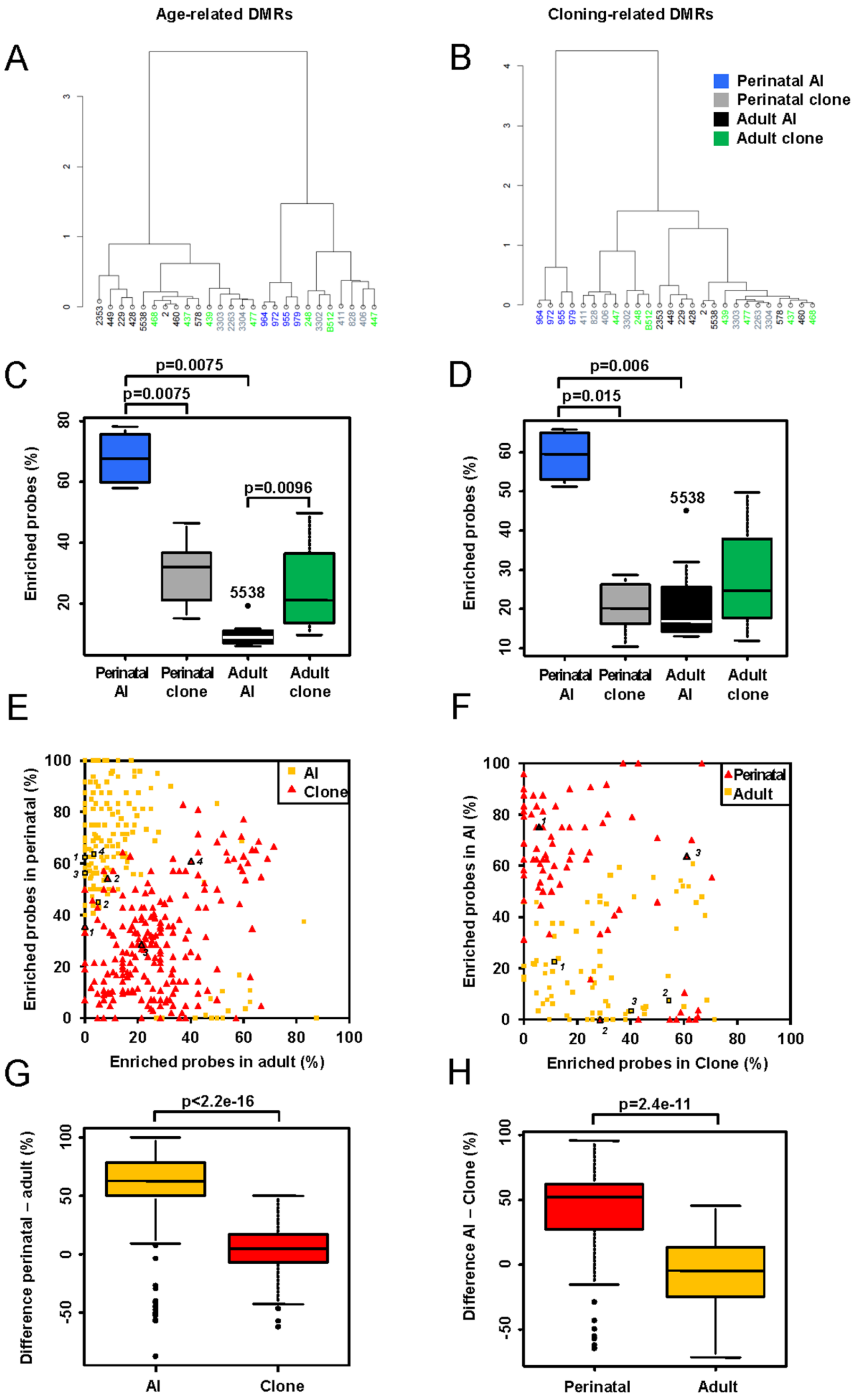
Age-related DMRs
Cloning-related DMRs
A
B
Perinatal AI
Perinatal clone
Adult AI
Adult clone
C
D
p=0.0075
p=0.0075
p=0.0096
p=0.006
p=0.015
5538
Enriched probes (%)
Perinatal AI
Perinatal clone
Adult AI
Adult clone
E
F
AI
Clone
Perinatal
Adult
Enriched probes in perinatal (%)
Enriched probes in adult (%)
Enriched probes in AI (%)
Enriched probes in Clone (%)
G
H
p<2.2e-16
p=2.4e-11
Difference perinatal – adult (%)
Difference AI – Clone (%)
AI
Clone
Perinatal
Adult

Supplementary Figure S12. Uncoupling of DNA methylation and age in pathological perinatal clones (previous page). (A, B) Hierarchical clustering on the number of enriched probes per DMR, for age-related (A) and cloning-related (B) DMRs. The y-axis indicates Pearson's correlation distance. (C, D) For each individual i the average percentage of enriched probes Pi was calculated considering all age-related DMRs (C) and all cloning-related DMRs (D). The boxplots show this percentage for perinatal AI (n=4), perinatal clones (n=7), adult AI (n=8) and adult clones (n=7). For each box, the middle line indicates the median and the edges the 25th/75th percentiles. Significant differences between groups are indicated ($p<0.05$, permutation test). (E) For each age-related DMR r, the percentage Pr of enriched probes in adults (x-axis) and perinatal animals (y-axis) were calculated separately for clones (red triangles) and AI controls (orange squares). Each DMR is therefore represented by two dots, whose relative distance reflects differences between clones and AI controls. The dots with black edges indicate the four regions shown in Supplementary Fig. S13. 1: DMR1; 2: DMR2; 3: DMR3; 4: DMR6. (F) For each cloning-related DMR r, the percentage Pr of enriched probes in clones (x-axis) and AI controls (y-axis) was calculated separately for perinatal animals (red triangles) and adults (orange squares). The dots with black edges indicate the three regions shown in Supplementary Fig. S13. 1: DMR4; 2: DMR5; 3: DMR6. (G) Boxplots showing the $Pr_{perinatal}$-Pr_{adult} difference (percentage of enriched probes in perinatal animals - percentage of enriched probes in adults) for each age-related DMR r, in AI controls (orange) and clones (red). The difference between the two groups is highly significant (Wilcoxon test for two paired samples). (H) Boxplots showing the Pr_{AI}–Pr_{clone} difference for cloning-related DMRs, in perinatal animals (red) and in adults (orange). The difference between the two groups is highly significant (Wilcoxon test for two paired samples).

A

Microarray DMR (microarray cohort)	CpGs analyzed by pyrosequencing	Correlation microarray-pyrosequencing	Differential methylation by pyrosequencing (microarray cohort)	Differential methylation by pyrosequencing (extended cohort)
DMR1	6	0.59	Yes (p=5e-04)	Yes (p=6e-05)
DMR2	9	0.76	Yes (p=7e-04)	Yes (p=4e-05)
DMR3	1	*0.31*	Yes (p=6e-04)	Yes (p=4e-05)
DMR4	6	0.45	*No (p=0.083)*	Yes (p=3e-03)
DMR5	22	0.68	Yes (p=5e-03)	Yes (p=3e-04)
DMR6	8	0.75	Yes (p=3e-04)	Yes (p=2e-05)
DMR7	5	0.39	Yes (p=8e-03)	*No (p=0.055)*
DMR8	9	*-0.07*	Yes (p=8e-04)	Yes (p=1e-05)
DMR9	30	0.53	Yes (p=5e-05)	Yes (p=2e-05)
DMR10	8	0.65	Yes (p=0.019)	Yes (p=6e-03)
DMR11	4	0.48	Yes (p=0.014)	Yes (p=1e-03)
DMR12	7	-0.49	Yes (p=0.023)	Yes (p=3e-03)

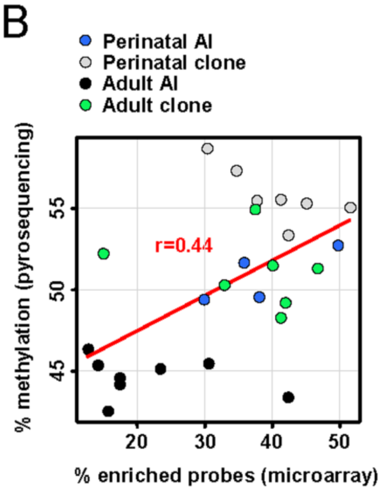

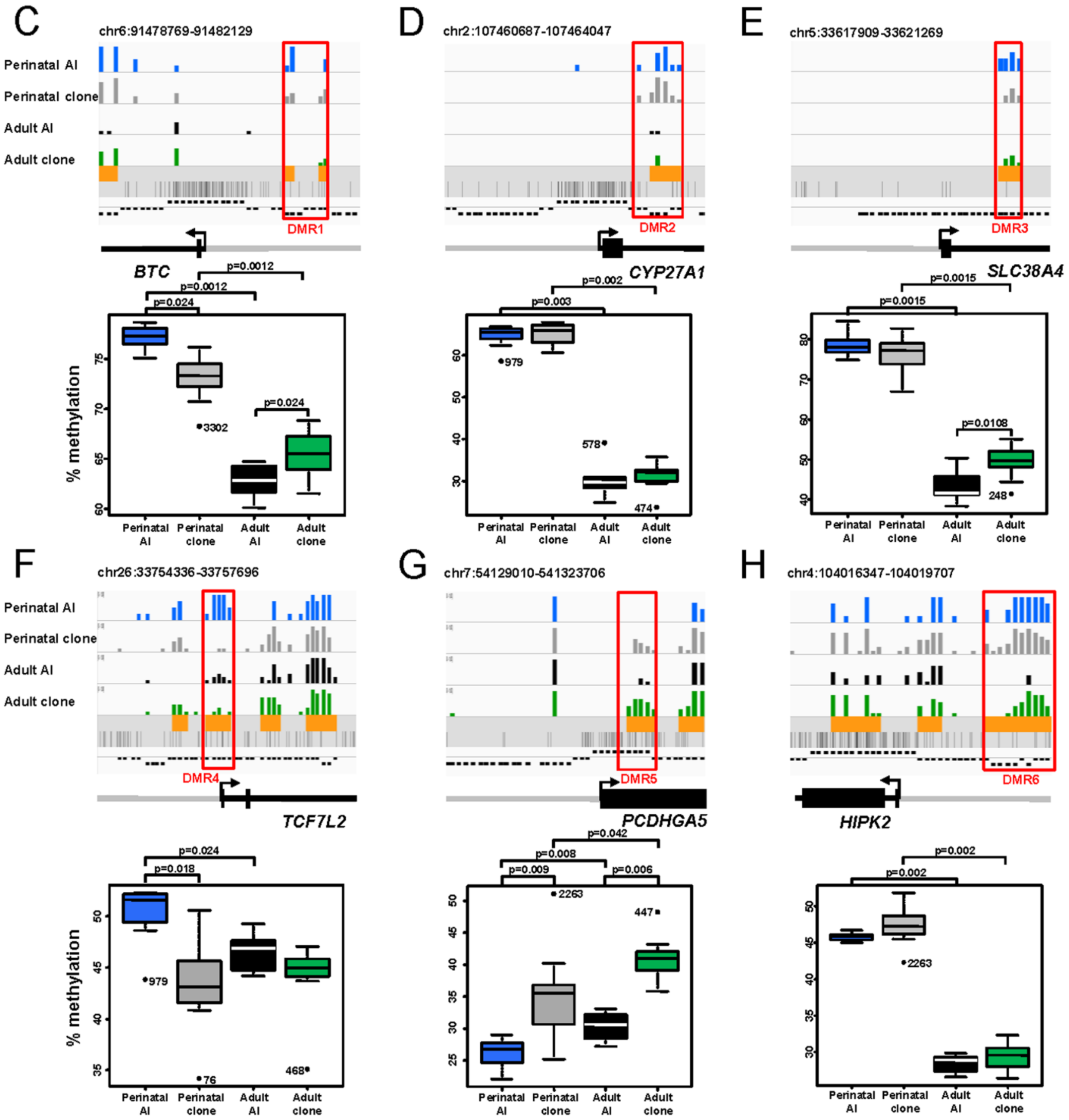

Supplementary Figure S13. Validation by pyrosequencing. (A) Table recapitulating the results obtained by pyrosequencing for 12 DMRs. For each DMR, the number of CpGs analysed, the correlation with microarray data (Spearman's rank correlation coefficients are underlined for significant correlations and displayed in italics otherwise), and the results of the statistical tests performed on pyrosequencing data for the microarray cohort (n=26) and the extended cohort (n=35) are shown. "Yes" indicates that the four groups were not identical (p<0.05; permutation test for k independent samples). (B) Correlation between the microarray data and the pyrosequencing results for the nine DMRs that were validated. Each dot represents one animal. The coordinates of each dot are given by the average methylation level assessed by pyrosequencing of 98 CpGs included in the nine regions (y-axis) and the percentage of enriched probes obtained from the microarray data on the same regions (x-axis). The least squares line of best fit and the Spearman's rank correlation coefficient r are shown in red for a significant correlation (Spearman's rank correlation test; p<0.05). (C-H) Microarray data and pyrosequencing results for DMR1-6. Upper panel: IGV browser views of the gene regions targeted by the microarray. The blue, grey, black and green bar charts represent the proportions of enriched probes at each probe position for the four groups of animals. Orange boxes indicate the regions of interest selected for differential analysis as defined in Supplementary Methods. CpGs are shown, as are the probe classes based on CpG frequency (the upper, middle and lower bands represent high, intermediate and low class probes, respectively). The red boxes delineate the regions analysed by pyrosequencing. Lower panel: average methylation level of the CpGs assayed by pyrosequencing for perinatal AI (n=7), perinatal clones (n=11), adult AI (n=8) and adult clones (n=9). Significant differences between groups are indicated (p<0.05, permutation test for pairwise comparisons).

A

Biological process	p-value	Genes
Transport	9.76E-03	*KCNA7, TBCEL, PKD2L1, SLC17A7, PITPNA, KCNQ4, AKR1A1, SEC23IP, SLC25A33, NFKBIB, SNAP47, MPZ, SLCO4C1, SCFD2, PAPPA2, BSP30C, SNUPN, SLC27A4, SLC7A7, RABAC1, SLC8A2, SAG, SCN11A, KIFC1, RTN2, PTH2R, SNX27, STX10*
Localization	1.35E-02	*KCNA7, TBCEL, PKD2L1, SLC17A7, PITPNA, KCNQ4, AKR1A1, SEC23IP, SLC25A33, NFKBIB, SNAP47, MPZ, SLCO4C1, SCFD2, PAPPA2, BSP30C, SNUPN, SLC27A4, SLC7A7, RABAC1, SLC8A2, SAG, SCN11A, KIFC1, RTN2, PTH2R, SNX27, STX10*
Glycogen metabolic process	2.30E-02	*CDKL4, G6PC3, PPP5C*
Lipid metabolic process	2.44E-02	*SLC17A7, SDR9C7, CYP27A1, SLC25A33, PITPNA, AMACR, NFKBIB, BSP30C, CHPT1, SLC27A4, ACSM1, INSIG2*
Cholesterol metabolic process	4.11E-02	*CYP27A1, BSP30C, INSIG2*

B

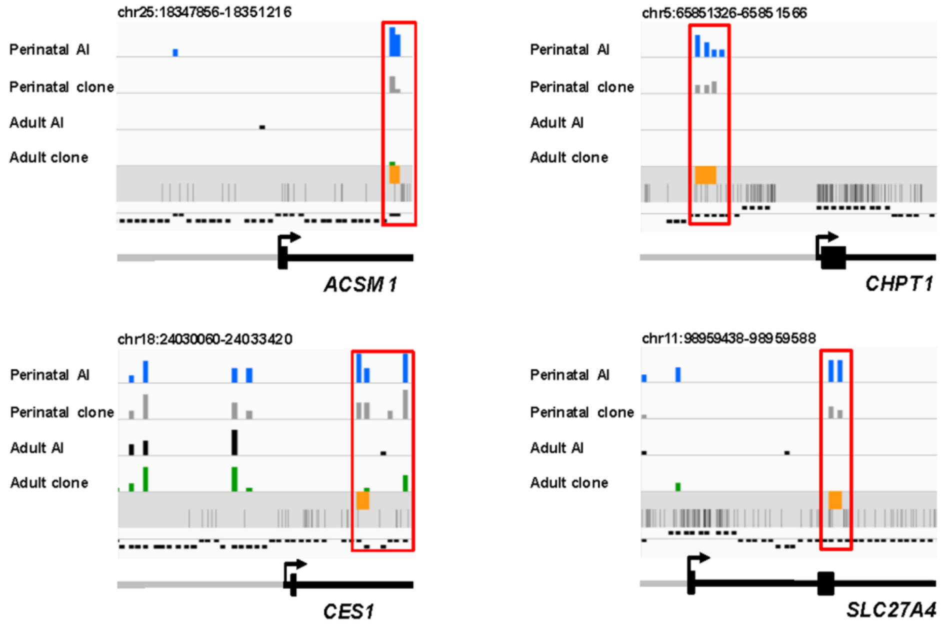

Supplementary Figure S14. DMRs associated with the normal transition to adult life target genes involved in glycogen, lipid and cholesterol metabolism. (A) GO analysis performed using PANTHER on the 182 DMRs significantly correlated to dimension 1 of MFA (Supplementary Table S6 and Figure 4). Terms of biological processes displaying a statistical over-representation among the DMRs are indicated, as are the corresponding genes. Genes shown in B or in Supplementary Fig. S13 are underlined. (B) IGV browser views showing regions targeted by the microarray for genes involved in lipid metabolism. DMRs are indicated by red boxes.

Supplementary Tables

Supplementary Table S1. Large regions and control region targeted by the microarray

Chromosomal coordinates	Genomic features	Number of probes included
chr1:155383445-155385421	*DAZL* exon 11, CpG poor	22
chr4:11907009-12704701	chr4 imprinted region	6779
chr4:95061962-95084691	*MEST* locus	261
chr6:37504213-37516145	*NAP1L5* locus	127
chr9:97633102-97746388	*IGF2R* locus	1184
chr13:58004925-58028709	*GNAS* locus	262
chr13:67113502-67125110	*NNAT* locus	156
chr18:64262266-64295800	*PEG3* locus	359
chr21:1-30936	*SNRPN* locus	319
chr29:49000000-50200000	chr29 imprinted region	11709
chrX:82256156-82299467	*XIST* locus	417
chrX:105375191-105467564	*MAOA* locus	792

Supplementary Table S2. Primers and PCR conditions used to generate the pyrosequencing templates

Primer name	Gene symbol (EMBL accession number)	Primer sequence (5'-3')	Size of the product	Hybridization temperature	$MgCl_2$ concentration	CpGs included
bBTC_bis_F1<br>bBTC_bio_R1	*BTC*<br>(ENSBTAG00000004237)	GGTGGGATTTGATGGTTTAGTATTA<br>CTATAAAAATAAACTTCTATATTAAAAAAA	219 bp	54°C	3 mM	2 (#1-2)
bBTC_bis_F2<br>bBTC_bio_R2	*BTC*<br>(ENSBTAG00000004237)	AAGTGATATTTTATTTTAAAATTAATTTA<br>TAAACCCTAAACTTCCTATACTCTTACC	236 bp	57.2°C	3 mM	4 (#3-6)
bCYP27A1_bis_F1<br>bCYP27A1_bio_F1	*CYP27A1*<br>(ENSBTAG00000013489)	GGTTGTGGTTATAATTTTGGA<br>AAAAAAAAATAATTCTATAACATCCC	214 bp	54.4°C	2 mM	5 (#1-5)
bCYP27A1_bis_F2<br>bCYP27A1_bio_R2	*CYP27A1*<br>(ENSBTAG00000013489)	ATTTAATGTTGGTTAAGTTGGAGAG<br>AAAAAAACATTTAACCCCCATAC	177 bp	57.9°C	3 mM	4 (#6-9)
bHIPK2_bis_F1<br>bHIPK2_bio_R1	*HIPK2*<br>(ENSBTAG00000017860)	TGAATTTATTTTTATTTTTTATTTTTTTT<br>AACTAATACTCCCCTTCCAAAC	289 bp	57.9°C	3.5 mM	3 (#1-3)
bHIPK2_bis_F2bis<br>bHIPK2_bio_R2bis	*HIPK2*<br>(ENSBTAG00000017860)	TGGATAGGAATGTTTTTTTAAAGGTAA<br>CAAAACACACAAATAAAAACATCAAA	153 bp	58°C	3 mM	2 (#4-5)
bHIPK2_bis_F3<br>bHIPK2_bio_R3	*HIPK2*<br>(ENSBTAG00000017860)	TTGTTTTTTTTGGAGTTAAGTTTT<br>AATCATAAAAATCAAATAACTAAATTTTTA	174 bp	58°C	3 mM	3 (#7-9)
bPCDHGA5_bis_F1<br>bPCDHGA5_bio_R1	*PCDHGA5*<br>(ENSBTAG00000045683)	GGTAGTTTAGTTATTGTAGATAGGATAGAT<br>TAAAAAAAATTCACACCCACATC	260 bp	60°C	3 mM	13 (#1-13)
bPCDHGA5_bis_F2<br>bPCDHGA5_bio_R2	*PCDHGA5*<br>(ENSBTAG00000045683)	AGTGGGAATATTATTAGTATTTTTTTT<br>ACCAATTCTAAATATTTCTATCCATCTC	144 bp	54°C	3 mM	4 (#11-14)
bPCDHGA5_bis_F3<br>bPCDHGA5_bio_R3	*PCDHGA5*<br>(ENSBTAG00000045683)	GAATTGGTATTGGAATAGTTTTTGG<br>CACTCACTCTATACTCAAATTAAATAAACA	175 bp	58°C	3 mM	8 (#15-22)
bSLC38A4_bio_F1<br>bSLC38A4_bis_R1	*SLC38A4*<br>(ENSBTAG00000014197)	ATTTTATTTTGGTGAAAGTTATTAGTATTA<br>TAATCAAACATTATACATACAATTC	110 bp	57.2°C	3 mM	1
bTCF7L2_bis_F1<br>bTCF7L2_bio_R1	*TCF7L2*<br>(ENSBTAG00000021574)	GAATTTTTAGTGGTTTTTGGGTATT<br>CCCTCAAACACAATTCTATTTTAAAA	206 bp	57.9°C	2 mM	3 (#1-3)
bTCF7L2_bis_F2<br>bTCF7L2_bio_R2	*TCF7L2*<br>(ENSBTAG00000021574)	TGAGAGTTTAGTTATTTAAATTTTGG<br>ACACCTATTCAATAAACAAAAAAAA	284 bp	57.9°C	3 mM	6 (#4-9)

The last column indicates the number of CpGs included in the PCR product. These CpGs are numbered from 5' to 3' according to their position along the chromosome.

Supplementary Table S3. Pyrosequencing primers

Name	Sequence (5'-3')	Gene symbol (EMBL accession number)	Template	CpG
bBTC_1_pyr0	TAAAGATAAAAGTAG	*BTC* (ENSBTAG00000004237)	bBTC_bis_F1 X bBTC_bio_R1	#1
bBTC_1_pyr1	GTAAGATTAATATGTG			#2
bBTC_bis_F2	AAGTGATATTTTATTTTAAAATTAATTTA	*BTC* (ENSBTAG00000004237)	bBTC_bis_F2 X bBTC_bio_R2	#3
bBTC_2_pyr1	TTTTATTTTAGTTTTTAT			#4
bBTC_2_pyr2	TTTTGGGAGGATAGG			#5-6
bCYP27A1_1_pyr0	TGGTTATAATTTTGG	*CYP27A1* (ENSBTAG00000013489)	bCYP27A1_bis_F1 x bCYP27A1_bio_R1	#1-2
bCYP27A1_1_pyr1	AGGAAGGGTATGAAG			#3-4
bCYP27A1_1_pyr2	TTGTAAGTGTGTAAG			#5
bCYP27A1_2_pyr0	AGGTTTTAGGAAGTG	*CYP27A1* (ENSBTAG00000013489)	bCYP27A1_bis_F2 x bCYP27A1_bio_R2	#6-8
bCYP27A1_2_pyr2	GGGAATTTTTTTAAG			#9
bHIPK2_bis_F1	TGAATTTATTTTTATTTTTTATTTTTTTT	*HIPK2* (ENSBTAG00000017860)	bHIPK2_bis_F1 x bHIPK2_bio_R1	#1
bHIPK2_1_pyr1	AGGTATTTAATATAG			#2
bHIPK2_1_pyr2	ATATTGTGTATATGG			#3
bHIPK2_2bis_pyr3	GTTTTTTTAGTAATTG	*HIPK2* (ENSBTAG00000017860)	bHIPK2_bis_F2bis x bHIPK2_bio_R2bis	#4-5
bHIPK2_bis_F3	TTGTTTTTTTTGGAGTTAAGTTTT	*HIPK2* (ENSBTAG00000017860)	bHIPK2_bis_F3 x bHIPK2_bio_R3	#7-8
bHIPK2_3_pyr1	GATGATTTTTTAAAG			#9
bPCDHGA5_1_pyr0	TTGTAGATAGGATAG	*PCDHGA5* (ENSBTAG00000045683)	bPCDHGA5_bis_F1 x bPCDHGA5_bio_R1	#1
bPCDHGA5_1_pyr01	GGGAGGAGTTTTG			#2-5
bPCDHGA5_1_pyr1	GGTAGAAGTAGAAAT			#6-9
bPCDHGA5_1_pyr2	AAGTTAATGAAAATG			#10
bPCDHGA5_bis_F2	AGTGGGAATATTATTAGTATTTTTTTT	*PCDHGA5* (ENSBTAG00000045683)	bPCDHGA5_bis_F2 x bPCDHGA5_bio_R2	#11-13
bPCDHGA5_2_pyr1	TTTGGATGAGAAAAG			#14
bPCDHGA5_3_pyr0	TGGAATAGTTTTTGG	*PCDHGA5* (ENSBTAG00000045683)	bPCDHGA5_bis_F3 x bPCDHGA5_bio_R3	#15-17
bPCDHGA5_3_pyr1	TATAGTTTTAGATGG			#18-22
bSLC38A4_bis_R1	TAATCAAACATTATACATACAATTC	*SLC38A4* (ENSBTAG00000014197)	bSLC38A4_bio_F1 x bSLC38A4_bis_R1	#1
bTCF7L2_1_pyr0	TGGATTTTTAGAGTG	*TCF7L2* (ENSBTAG00000021574)	bTCF7L2_bis_F1 x bTCF7L2_bio_R1	#1-2
bTCF7L2_1_pyr1	AAAGAATGTATAAGG			#3
bTCF7L2_2_pyr0	TTATTTAAATTTTGG	*TCF7L2* (ENSBTAG00000021574)	bTCF7L2_bis_F2 x bTCF7L2_bio_R2	#4
bTCF7L2_2_pyr2	TTATGTTTTAATGGG			#8-9

The last column indicates the CpG(s) analyzed with each primer.
